# Opto‐Lipidomics of Tissues

**DOI:** 10.1002/advs.202302962

**Published:** 2023-12-25

**Authors:** Magnus Jensen, Shiyue Liu, Elzbieta Stepula, Davide Martella, Anahid A. Birjandi, Keith Farrell‐Dillon, Ka Lung Andrew Chan, Maddy Parsons, Ciro Chiappini, Sarah J. Chapple, Giovanni E. Mann, Tom Vercauteren, Vincenzo Abbate, Mads S. Bergholt

**Affiliations:** ^1^ Centre for Craniofacial and Regenerative Biology King's College London London SE1 9RT UK; ^2^ Institute of Pharmaceutical Science King's College London London SE1 9NH UK; ^3^ King's British Heart Foundation Centre of Research Excellence School of Cardiovascular and Metabolic Medicine & Sciences Faculty of Life Sciences & Medicine King's College London 150 Stamford Street London SE1 9NH UK; ^4^ Randall Centre for Cell and Molecular Biophysics King's College London London SE1 1UL UK; ^5^ School of Biomedical Engineering and Imaging Sciences King's College London London WC2R 2LS UK; ^6^ Department of Analytical Environmental and Forensic Sciences King's College London 150 Stamford Street London SE1 9NH UK

**Keywords:** lipidomics, mass spectrometry, Raman spectroscopy

## Abstract

Lipid metabolism and signaling play pivotal functions in biology and disease development. Despite this, currently available optical techniques are limited in their ability to directly visualize the lipidome in tissues. In this study, opto‐lipidomics, a new approach to optical molecular tissue imaging is introduced. The capability of vibrational Raman spectroscopy is expanded to identify individual lipids in complex tissue matrices through correlation with desorption electrospray ionization (DESI) – mass spectrometry (MS) imaging in an integrated instrument. A computational pipeline of inter‐modality analysis is established to infer lipidomic information from optical vibrational spectra. Opto‐lipidomic imaging of transient cerebral ischemia‐reperfusion injury in a murine model of ischemic stroke demonstrates the visualization and identification of lipids in disease with high molecular specificity using Raman scattered light. Furthermore, opto‐lipidomics in a handheld fiber‐optic Raman probe is deployed and demonstrates real‐time classification of bulk brain tissues based on specific lipid abundances. Opto‐lipidomics opens a host of new opportunities to study lipid biomarkers for diagnostics, prognostics, and novel therapeutic targets.

## Graphical abstract

In the study, the capability of Raman spectroscopy is expanded to identify lipids in complex tissue matrices through correlation with desorption electrospray ionization (DESI) ‐ mass spectrometry (MS) imaging in an integrated instrument. Specific lipid imaging is demonstrated in a murine model of ischemic stroke, and the technique is deployed in a fiber‐optic Raman probe for in situ lipidomics.

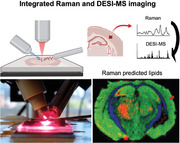

## 1 Introduction

Lipids, which include triglycerides, phospholipids, sterols, fatty acids, polyketides, and saccharolipids are essential molecules providing fundamental building blocks of cells and tissues. Lipids are key components of plasma membranes, and the makeup of these membranes affects many biological functions, such as enzyme regulation and receptor activity. Additionally, certain types of lipid structures, such as lipid rafts, have crucial roles in maintaining proper cellular activity and function. Many diseases, including ischemic stroke, atherosclerosis, neurological diseases, and cancers, are all associated with dysregulation in lipid content.^[^ ^1^, ^2^ ^]^

Although lipids play a crucial role in many biological processes, imaging strategies to investigate them are limited since they are not amenable to labeling with fluorophores. To perform lipidomic analyses, scientists currently depend heavily on high‐performance liquid chromatography (HPLC) and mass spectrometry (MS) of bulk tissues.^[^ ^3^ ^]^ MS enables the identification and quantification of molecules based on their mass‐to‐charge ratio (m/z). Lipids are extracted and isolated from the complex tissue matrix and transformed into charged ions using techniques such as electrospray ionization (ESI) and matrix‐assisted laser desorption/ionization (MALDI) for mass analysis.

Spatial lipidomics approaches based on MS provide an unprecedented opportunity to study how information at the molecular scale contributes and shapes tissue functions and phenotype. In particular, desorption electrospray ionization (DESI)‐MS has emerged as a powerful ambient approach for conducting spatial lipidomics of tissue samples.^[^ ^4^ ^]^ Optical vibrational techniques such as Fourier transform – infrared (FT–IR) or Raman spectroscopy can also be used for analysis of lipids.^[^ ^5^ ^]^ Raman spectroscopy uses laser light to probe specific vibrational modes associated with the structure of molecules.^[^ ^6^ ^]^ Raman spectroscopy holds great appeal for tissue analysis both ex vivo and in vivo due to its non‐destructive nature, label‐free detection scheme, and minimal sample preparation requirements.^[^ ^7^ ^]^

Optical spectroscopy and MS generally provide complementary information about tissue composition.^[^ ^8^ ^]^ DESI‐MS – typically involving high‐resolution MS, offers precise and untargeted detection and identification of lipids and metabolites based on mass‐to‐charge ratios and determination of elemental composition. Raman spectroscopy probes the specific molecular vibrations in proteins, lipids, carbohydrates, and DNA, enabling the analysis of subtle structural molecular differences.^[^ ^5^ ^]^ While Raman spectroscopy can uncover the overall lipid content, it remains extremely difficult to distinguish different lipid subtypes in tissue matrices. This complexity arises due to the overlapping Raman peaks from a myriad of molecules in tissues, resulting in a compound tissue spectrum.^[^ ^9^ ^]^ This presents a significant challenge for any vibrational spectroscopy technique in the field of biomedical sciences. Raman spectroscopy has, however, unexploited potential to offer massively multiplexed tissue analytics ex vivo and in vivo. Existing methods for analyzing compound tissue spectra rely predominantly on multivariate regression analysis using libraries of purified chemicals, principal component analysis (PCA)^[^ ^10^ ^]^ or clustering of molecular fingerprints.^[^ ^11^ ^]^ Although these computational techniques are effective in estimating the overall molecular composition (e.g., total protein, lipid, and DNA content), they often fall short in enabling precise identification and subtyping of lipids in tissues.^[^ ^12^ ^]^ Sequential imaging using optical spectroscopy and MS has therefore been reported for tissue analysis.^[^ ^8^, ^13^ ^]^ The utilization of both Raman and MS data enhances tissue characterization due to their complementary nature.

Here we introduce a new strategy for optical lipid imaging. We expand the capability of vibrational Raman spectroscopy to identify individual lipids in complex tissue matrices. Development of the first integrated Raman and DESI‐MS instrument enabled us to perform fully correlative lipid imaging of tissues. This allowed us to develop a multivariate model for inferring lipidomic information from optical vibrational spectra. We demonstrate the use of this method, “opto‐lipidomics”, by imaging ischemia‐reperfusion brain injury in a murine model of transient ischemic stroke showing that Raman spectroscopy can detect and distinguish specific lipids in complex tissues. Finally, we deploy opto‐lipidomics in a handheld fiber‐optic Raman probe demonstrating real‐time bulk tissue lipidomics. This optical method achieves, for the first time, direct label‐free visualization, and detection of specific lipid subtypes in complex tissues.

## 2 Results

### 2.1 Integrated Raman/DESI‐MS Imaging

Sequential optical spectroscopy and MS imaging of tissues present substantial technical challenges, including the need for precise image resolution matching, sampling spot area matching, absolute pixel‐to‐pixel alignment, diverse sample preparation methodologies, and slow imaging that result in degradation or evaporation of volatile compounds from the tissue (Figure S1, Supporting Information). Furthermore, achieving co‐registration and correlation of data from various modalities with distinct types of molecular information presents a challenging task, in particular when the information is hidden in compounded Raman peaks. Raman spectroscopy and DESI‐MS imaging, however, exhibit remarkable compatibility, given that they both utilize ambient label‐free detection schemes. Integrating these modalities into a single instrument could overcome many of these key challenges, but it mandates a thorough evaluation.

**Figure 1:**
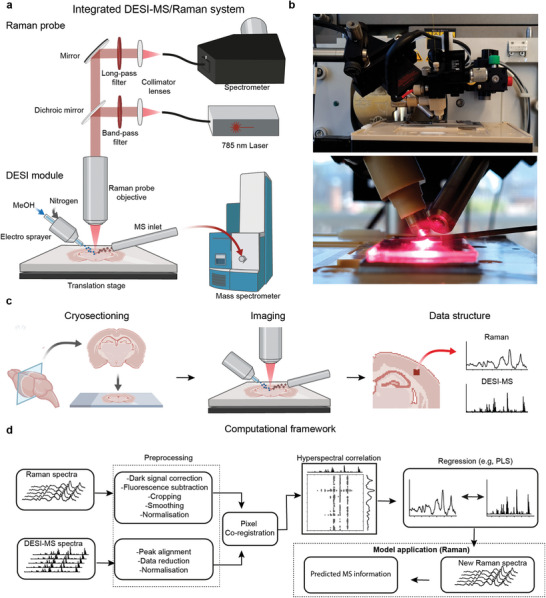
Integrated Raman and DESI‐MS imaging system. a) Schematics of the integrated Raman and DESI‐MS imaging system. The electro‐sprayer pneumatically focuses electrically charged MeOH solvent on the tissue sample using pressurized nitrogen. Desorbed molecules are then passed through ambient air into the mass spectrometer inlet. For Raman spectroscopy, a 785 nm laser light is delivered through an objective lens and focused onto the sample at a 45‐degree angle. Raman scattered light is passed back through the objective and separated from the laser path by a dichroic mirror and long pass filter before it is fiber‐coupled into a near‐infrared (NIR) Raman spectrometer. b) Photograph showing the integrated Raman/DESI‐MS imaging system using a commercial Raman probe (InPhotonics). c) Integrated Raman/DESI‐MS imaging workflow for tissue analysis including cryosectioning, correlative imaging, and analysis. d) Computational framework for analysis: Data processing and correlation analysis pipeline for Raman and DESI‐MS data. The Raman and DESI‐MS data are preprocessed separately before they are co‐registered. Heterospectral analysis is performed to ensure accurate co‐registration and can be used to confirm their correlation. Lastly, a regression model can be constructed to predict relative m/z abundances from the Raman spectra.

We developed an integrated Raman/DESI‐MS imaging instrument with closely matched spatial resolution (**Figure** **1**a–c). The spatial resolution for correlative imaging is limited to ≈50 µm by DESI‐MSI. Due to the less tightly focused Raman laser excitation, the laser power density was substantially lower (<10×) than for a typical Raman microscope reducing the risk of local tissue heating. Raster scanning is used to acquire data from the tissue and is controlled using an in‐house developed software suite to enable electronic synchronization with the motorized stage (Figure S2, Supporting Information). We verified that the electrically charged MeOH jet spray did not give rise to interfering Raman peaks for any flow rates (Figure S3, Supporting Information). This is due to the comparatively low detection sensitivity of Raman spectroscopy. We created protocols for Raman/DESI‐MS imaging alignment and substrate preparation (see Experimental Section). Since Raman spectra are sampled at an angle, we characterized and found only subtle angular dependence in the tissue Raman signal intensity (Figure S4, Supporting Information). The characterization of the impact of tissue thickness on Raman signal generation indicated that a thickness of 40 µm (corresponding to approximately three times a cell size) provided a reasonable compromise between tissue thickness and signal intensity generation (Figure S5, Supporting Information). DESI‐MS imaging can be considered a partially destructive technique due to the exposure of tissue to MeOH and desorption/ionization of the superficial tissue layer (Figure S6, Supporting Information). Therefore, we investigated the impact of DESI‐MS imaging on the Raman signal. However, we found no major differences induced by DESI‐MS, as revealed by PCA suggesting these are subtle or below the detection limit (Figure S7, Supporting Information). The integration of Raman and DESI‐MS imaging produces two extensive hyperspectral datasets necessitating processing. We, therefore, created a computational pipeline for handling the two modalities (Figure 1d), including background image masking, separate preprocessing for Raman spectra (Figure S8, Supporting Information) and DESI‐MS spectra, pixel‐wise co‐registration hetero‐spectral correlation analysis, and multivariate regression (see Experimental Section).

We investigated the complementarity between Raman spectroscopy and DESI‐MS by measuring structural isomers bearing identical elemental composition that cannot be differentiated using DESI‐MS alone (Figure S9, Supporting Information). These results demonstrate that while DESI‐MS can provide specific identification of lipid sub‐classes, Raman spectroscopy can probe subtle differences in molecular structure and distinguish highly similar lipids. In a controlled mixture experiment involving two lipids (PC18:1‐18:0(1‐oleoyl‐2‐stearoyl‐sn‐glycero‐3‐phosphocholine) and L‐α‐Phosphatidylcholine as reference), we observed that Raman spectroscopy demonstrated linearity with concentration while DESI‐MS exhibited a lower degree of linearity for this arbitrarily chosen mixture (Figure S10, Supporting Information).

### 2.2 Opto‐Lipidomic Imaging of Mouse Brain Tissues

**Figure 2:**
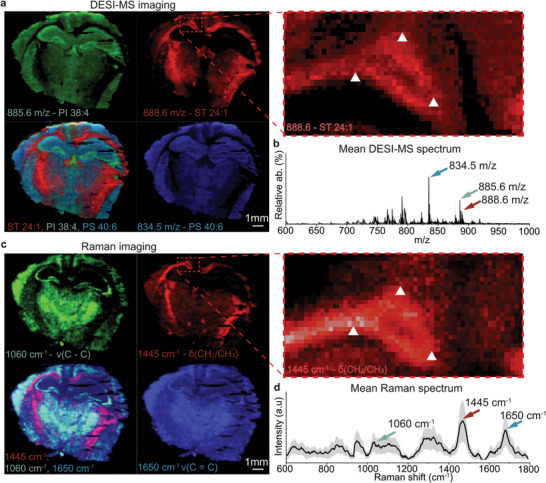
Co‐registered Raman/DESI‐MS imaging of healthy mouse brain tissue. Co‐registration of the Raman and DESI‐MS hyperspectral images both sampled at 50 µm resolution. a) DESI‐MS image of PI 38:4, ST 24:1, and PS 40:6. b) Mean DESI‐MS spectrum of the entire brain tissue. c) Raman images generated from peaks at 1060, 1445, and 1650 cm^−1^. White triangles in DESI‐MS and Raman images indicate markers of similar anatomical location. d) Mean Raman spectrum ± 1 standard deviation (SD) of all tissue spectra from the brain.

Using the integrated Raman/DESI‐MS imaging instrument, we imaged 8 coronal tissue sections from the brains of healthy mice as these exhibit well‐defined anatomical landmarks of white and grey matter (384000 Raman spectra and 384000 DESI‐MS spectra) (**Figure** **2**a–d; Figure S11, Supporting Information). DESI‐MSI false‐color images allow visualization of the presence of prominent lipids and their relative abundances tentatively assigned using MS/MS (Tables S1 and S2, Supporting Information): PI 38:4 (m/z 885.6), ST 24:1 (m/z 888.6), and PS 40:6 (m/z 834.5) associated with white and grey matter (Figure 2a). The mean DESI‐MS spectrum for the entire tissue section showed distinct m/z peaks previously reported (Figure 2b).^[^ ^14^ ^]^ Correlation analysis between m/z peaks indicated that the samples contained peaks that were independently distributed in the tissue to various degrees (Figure S12, Supporting Information). In contrast to DESI‐MS, which offers molecular identification, the average Raman spectra of tissues contained numerous overlapping peaks, resulting in limited molecular specificity (Figure 2c,d). However, by imaging prominent Raman peaks, such as 1445 cm^−1^ (CH_2_ deformation of lipids), 1650 cm^−1^ (υ(C═C) of lipids + Amide I of proteins) and 1060 cm^−1^ (tentatively assigned to DNA), we observed similar anatomical landmarks.

**Figure 3:**
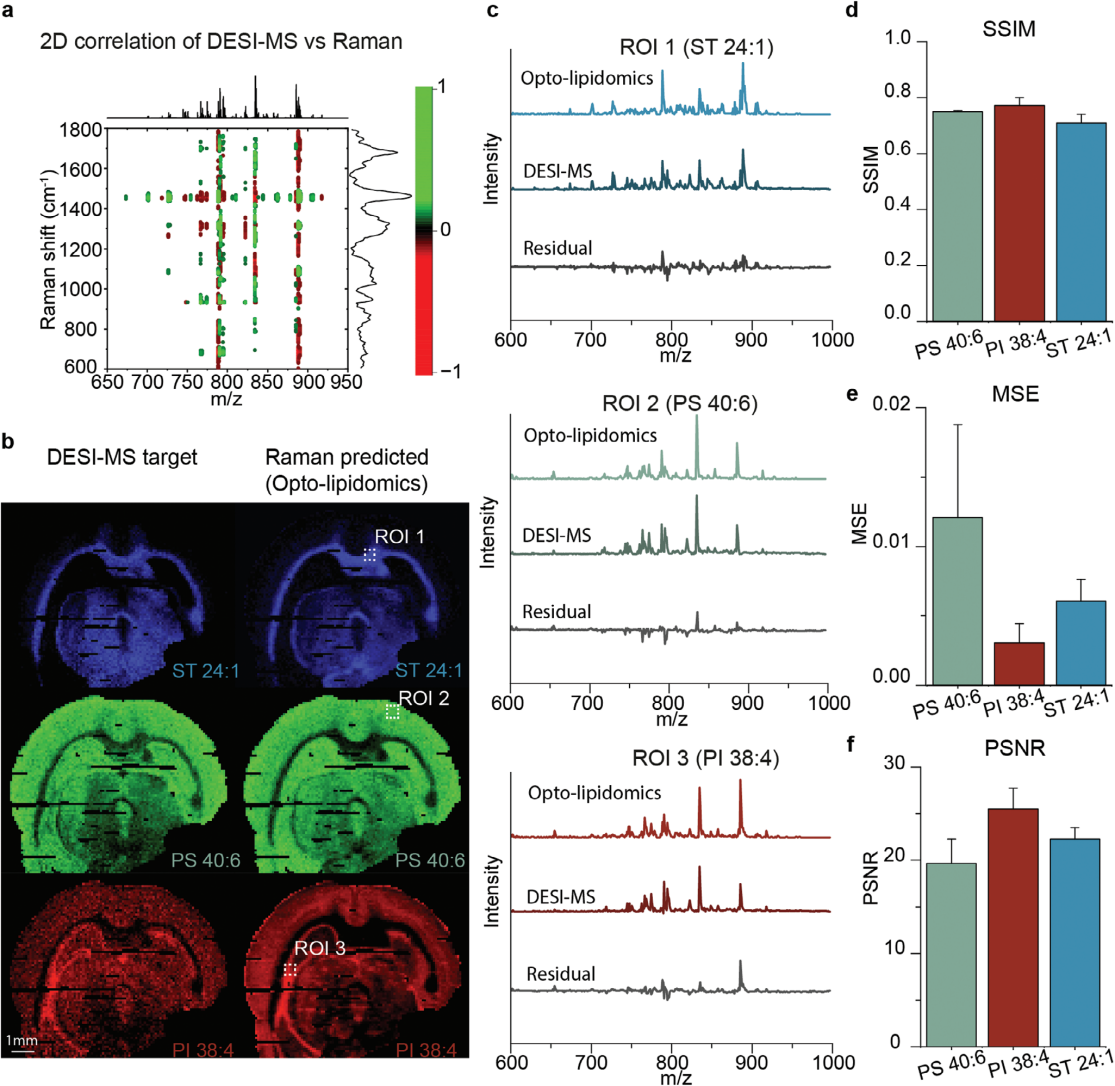
Opto‐lipidomic imaging of healthy mouse brain tissue. a) 2D heterospectral correlation between Raman and DESI‐MS for healthy mouse brain tissue. b) Images of a coronal mouse brain section showing target DESI‐MS and Raman predicted (opto‐lipidomics) images: ST 24:1, PS 40:6, and PI 38:4. c) Representative mean spectra of region of interest (ROI) associated with the three lipids using DESI‐MSI and Raman predicted (opto‐lipidomics). Also shown are the residual spectra. Image quality metrics comparing Raman predicted versus DESI‐MS imaging: d) Structural similarity index (SSIM), e) Mean squared error (MSE), and f) peak signal to noise ratio (PSNR), for PS 40:6, PI 38:4, and ST 24:1 (n = 3) (error bars: mean ± standard deviation (SD)).

Heterospectral correlation analysis demonstrated a weak but complex yet interpretable relationship between tissue Raman peaks and MS lipid abundances (**Figure** **3**a). For example, the positive correlation between the 1445 cm^−1^ Raman peak (CH_2_ deformation of lipids) and ST 24:1 (m/z 888.6) confirmed why Raman spectroscopy provides a contrast of white matter.

Conventional PCA analysis of the brain tissue Raman images offers limited insights into the tissue's lipidomics (Figure S13, Supporting Information). Our goal was to expand the capability of Raman spectroscopy to identify individual lipids in complex tissue matrices through correlation with DESI‐MS imaging. We, therefore, performed inter‐modality partial least squares (PLS) regression analysis to predict the relative m/z abundances from the Raman spectra. The developed regression model was used to predict the m/z abundances in 3 sections from one independent mouse brain (70389 Raman spectra and 70389 DESI‐MS spectra). We imaged the distribution of three of the most abundant m/z peaks as well as the Raman predicted (opto‐lipidomic) distributions (Figure 3b). The Raman‐predicted lipid distribution images showed striking similarities with DESI‐MSI, such as a nearly indistinguishable distribution of ST 24:1 associated with myelinated tissues and PS 40:6 of grey matter . The average DESI‐MS spectrum and the Raman‐predicted (MS) spectrum for the three regions of interest exhibited a strong correlation (Figure 3c). The residual difference spectra also reveal that the model exhibited poor performance for certain m/z peaks (e.g., m/z 794.6) (Figure S14, Supporting Information). The images of the 12 most prominent DESI‐MS peaks and their predicted lipid distributions can be found in Figure S15 (Supporting Information). We quantitatively compared opto‐lipidomics and DESI‐MS imaging for various m/z peaks. We calculated the structural similarity index (SSIM)^[^ ^15^ ^]^ (Figure 3d), mean square error (MSE) (Figure 3e), and peak signal‐to‐noise ratio (PSNR) (Figure 3f). The congruence between the predicted and observed lipid distributions confirms the feasibility of extracting lipidomics information from optical vibrational spectra using DESI‐MS.

### 2.3 Opto‐Lipidomic Imaging in a Murine Model of Transient Cerebral Ischemia‐Reperfusion Injury

**Figure 4:**
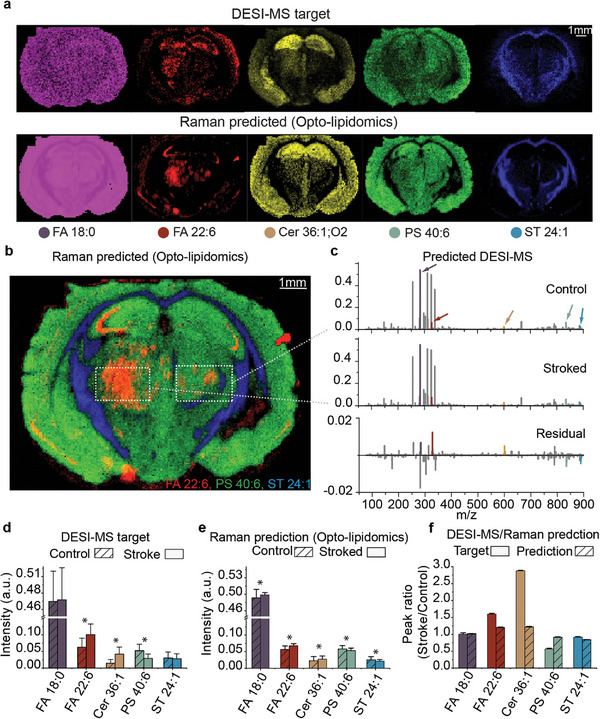
Opto‐lipidomics in a mouse model of transient cerebral ischemia‐reperfusion injury. a) Images of a coronal mouse brain section post ischemia‐reperfusion showing DESI‐MSI target and Raman predicted (opto‐lipidomic) images associated with the brain (FA 18:0, FA 22:6, Cer 36:1; O2, PS 40:6, and ST 24:1. b) Merged Raman predicted (opto‐lipidomic) distribution of FA 22:6, PS 40:6, and ST 24:1. c) Representative mean predicted MS spectra for the stroked and contralateral control side. Also shown is the residual. d) DESI‐MS target peak intensity values in stroked and control side ROI for FA 18:0, FA 22:6, Cer 36:1; O2, PS 40:6, and ST 24:1. e) Raman predicted (opto‐lipidomic) peak intensity values in stroked and normal side ROI for FA 18:0, FA 22:6, Cer 36:1;O2, PS 40:6, and ST 24:1. f) DESI‐MS target and Raman predicted peak residual values between stroked and normal side ROI for FA 18:0, FA 22:6, Cer 36:1;O2, PS 40:6, and ST 24:1. (Data denote mean ± standard deviation (SD), ^*^ = Significant difference between stroked and control side, *p* < 0.05).

We next investigated if opto‐lipidomic imaging could be used to identify changes in lipids in a disease model. Ischemic stroke is a leading cause of mortality and adult disability, and restoration of cerebral blood flow is currently the only effective treatment. Cerebral ischemia/reperfusion injury is associated with increased generation of reactive oxygen species,^[^ ^16^ ^]^ mitochondrial dysfunction,^[^ ^17^, ^18^ ^]^ and dysregulation of lipid metabolism,^[^ ^19^ ^]^ yet studies of altered lipid metabolism in ischemic stroke are limited. We imaged 3 tissue sections from a mouse model of transient cerebral ischemia‐reperfusion injury, collecting 77032 Raman spectra and 77032 DESI‐MS spectra. In this model, one‐sided reperfusion injury was induced, while the contralateral brain hemisphere served as internal control tissue. Regression analysis was used to predict the m/z abundances in an independent mouse brain section with one‐sided reperfusion injury (**Figure** **4**a–c; Figure S16, Supporting Information). These results show that opto‐lipidomic imaging largely replicated the DESI‐MSI results. Region‐of‐interests (ROIs) in injury and control side (Figure 4b,c) revealed that FA 22:6 (m/z 327.2 associated with docosahexaenoic acid (DHA)) and CER 36:1;O2 (m/z 600.5) were accumulated in the injured hemisphere. Most importantly, these Raman‐derived observations from the ROIs mostly agreed with the conclusions drawn from DESI‐MSI (Figure 4d–f). Image quality metrics (SSIM, PSNR, and MSE) revealed that the predicted lipid distributions recapitulate the DESI‐MSI findings to various degrees (Figure S17, Supporting Information).

### 2.4 Handheld Real‐Time Opto‐Lipidomics of Bulk Tissues

**Figure 5:**
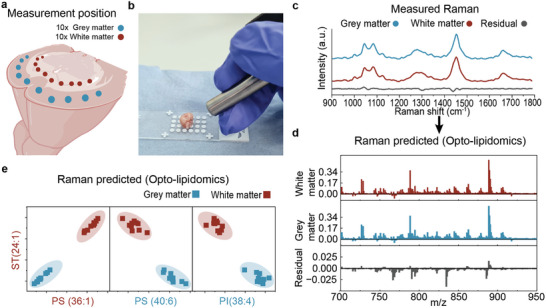
Handheld real‐time opto‐lipidomics of bulk tissues. a) Schematic of coronal cut mouse brain with measurement position represented in blue (grey matter) and red (white matter). b) Image of handheld Raman probe and coronal cut mouse brain. c) Representative mean Raman spectra for the grey matter, white matter as well as the residual difference spectrum. d) Representative mean predicted DESI‐MS spectra for white matter and grey matter. Also shown is the residual. e) Scatter plot depicting the predicted content of PS 36:1, PS 40:6, PI 38:4, against ST 24:1, showing separation of white and grey matter measurements (n = 20).

As a final demonstration, we obtained an intact mouse brain and excised it in the transverse plane (**Figure** **5**a). We then used a handheld fiber‐optic Raman probe for real‐time (0.5 s acquisition) sampling across the white and grey matter using the same spectrometer to preserve the multivariate calibration (Figure 5b). The model previously trained on (77032 Raman and 77032 DESI‐MS spectra) was used to predict the m/z abundances in bulk tissue. We acquired Raman spectra of normal mouse brains containing visually apparent white matter (10 spectra) and grey matter (n = 10 spectra) (Figure 5c). Due to diffuse light scattering in bulk tissues, there were only subtle differences in the Raman spectra of white and grey matter (Figure 5c; Figure S18, Supporting Information). Nevertheless, opto‐lipidomic predictions of lipid abundances (ST 24:1 vs PS 36:1, ST 24:1 vs PS 40:6, and ST 24:1 vs PI 38:4) still demonstrated 100% discrimination between white and grey matter (Figure 5e). These results highlight the first proof of concept of a workflow for opto‐lipidomics of bulk tissues.

## 3 Discussion

Currently, there are limited optical tools that can directly visualize the lipidome in tissues. Here, we presented a new approach that can generate optical contrast of specific lipids. While conventional Raman spectroscopy is sensitive to lipids, its ability to accurately identify lipid species within complex tissue matrices is inherently limited. We have demonstrated that Raman spectroscopy and DESI‐MS complement each other in their capacity to analyze lipids and that they can be sensitive to some of the same molecules. We showed that this can be harnessed for inferring lipidomics from complex tissue Raman spectra. To achieve this, we engineered an integrated Raman and DESI‐MS imaging instrument enabling us to collect large datasets, reduce sample degradation, and enable precise sampling and co‐registration.

We demonstrated the application of opto‐lipidomics using healthy and diseased mouse brain tissue because the anatomical landmarks are well‐defined. For many of the most abundant DESI‐MS peaks, the Raman prediction and image reconstruction excellently replicated the DESI‐MS m/z abundances. However, we found that the predictive capability was reduced for some lower‐abundance DESI‐MS peaks (Figure S15, Supporting Information). For instance, we observed that in some cases Raman spectroscopy hallucinated lipids such as m/z 701.6 and m/z 766.6 (Figure S15, Supporting Information). This is expected and might be attributed to several limiting factors including low detection sensitivity of Raman spectroscopy, low MS signal, the presence of MS fragments/adducts and isomers, as well as the general inadequacy of linear regression modeling. Raman spectroscopy and DESI‐MS are both expected to have limitations in terms of quantitative analysis in tissues. However, they both exhibit varying degrees of complex proportionality with molecular abundances. Here we employ PLS regression since it offers straightforward model interpretability. We anticipate that employing more advanced non‐linear computational methods would further enhance imaging capabilities.

Application of opto‐lipidomics to a murine model of transient cerebral ischemia‐reperfusion injury revealed a significant increase of FA 22:6 (DHA) in the stroke hemisphere that has previously been associated with neural protection.^[^ ^20^ ^]^ Docosanoids have been shown to increase neurogenesis, angiogenesis, and, importantly, synthesis of one of its derivatives, neuroprotection D1 in the penumbra of the stroke hemisphere, thereby potentially affording protection against further ischemic damage. Moreover, pro‐inflammatory ceramide 36:1;O2 (CER) was also shown to accumulate within the stroked hemisphere, with previous studies showing a correlation between plasma CER accumulation and stroke risk and severity.^[^ ^21^ ^]^ These findings showcase the utility of opto‐lipidomics in investigating lipid biomarkers for applications in studying disease and potentially identifying therapeutic targets.^[^ ^22^ ^]^

Handheld opto‐lipidomics of bulk tissues served as an important proof of concept of how lipidomic information can be extracted optically. This is in sharp contrast to state‐of‐the‐art computational methods for Raman spectroscopy, such as PCA, which does not offer specific molecular identification (Figure S13, Supporting Information). Given that Raman spectroscopy can be performed in humans,^[^ ^23^ ^]^ the developed approach opens the door for in vivo optical lipidomics. Applications in living tissues, however, would necessitate rigorous validation (i.e., due to hydration/vascularity) in vivo.

The imaging methodology is subject to important limitations. Spectral normalization is necessary for both Raman and DESI‐MS imaging to enable comparative analysis between images. Further, if the tissue is highly homogenous or if multiple lipid species covariates, this can potentially lead to deceitful analysis. However, we anticipate that the most recent development of DESI‐MS sprayers with imaging resolution down to <10 micrometers will allow us to uncover much more details of the extracellular matrix and even offer single‐cell opto‐lipidomics which will likely improve analysis of more heterogenous tissues. Interestingly, recent development in DESI‐MS has shown the capability of imaging protein/peptides using tissue digestion techniques, significantly expanding the potential of this methodology.^[^ ^24^ ^]^ It should also be noted that the regression model was developed for a particular tissue type and thus needs to be validated prior to implementation for other applications. Our future objective is to introduce deep learning‐based analysis and transfer learning approaches, which will facilitate the expansion of the modeling capability to additional tissues and systems with minimal effort.^[^ ^25^ ^]^

Many prospects exist for improving the predictive capability of the imaging approach. Due to various experimental factors, such as total laser power, sample dimensions, electrospray flow rate, and the size of the electro‐sprayer reservoir, the minimum integration time per pixel was determined to be approximately ≈0.5 s. Extending the integration time may enhance the detection limit of Raman spectroscopy but will also lead to longer imaging times. DESI‐MS analyses a superficial tissue layer, whereas Raman spectroscopy, utilizing NIR laser excitation, probes the entire 40 µm tissue thickness. Therefore, developing specialized focusing optics with extended working distance and genuine confocal capability could potentially enhance the correlation even more. Additionally, since Raman spectroscopy can be diffraction limited it might be possible to predict subcellular components, however, the validation of this will be challenging with the limited resolution of DESI‐MSI. Exploring avenues for subcellular or functional imaging at molecular, cellular, and tissue levels might therefore also involve correlating high‐resolution Raman spectroscopy with complementary analyses such as immunofluorescence, proteomics, and spatial transcriptomics.

## 4 Conclusion

We developed a unified Raman and DESI‐MS imaging instrument. This enabled us to expand the capability of vibrational Raman spectroscopy to identify individual lipids in tissues. This creates novel possibilities for tissue characterization and highlights a new strategy for implementing optical techniques in the imaging and detection of lipids.

## 5 Experimental Section

### Development of Integrated Raman and DESI‐MSI Instrument

In DESI‐MSI, an electro‐sprayer pneumatically focuses a jet of electrically charged MeOH solvent onto the tissue sample using pressurized nitrogen. The DESI‐MS system (Waters Corporation) comprises a DESI module interfaced to a high‐resolution hybrid quadrupole‐time of flight mass spectrometer (XEVO G2‐XS QTOF MS) operated in sensitivity mode (Negative ion, MS resolution: 2000, Window: 0.02). The DESI module encompasses a sprayer head and a motorized sample stage. Nebulizing nitrogen gas was externally supplied to the system. An electrospray solvent (HPLC‐grade MeOH, ≥99.9%, Sigma Aldrich 67‐65‐1) was passed to the electro‐sprayer head through a fused silica capillary tube (Waters 6490512‐S3) attached to a 10 ml syringe (SGE Model 1MR‐GT 1 ml/23/2). The syringe was placed into a syringe pump (Harvard Apparatus, Pump 11 Elite), which applied pressure to the syringe, slowly supplying a constant flow of solvent to the sprayer head. The sample stage controller was connected to the mass spectrometer controlled by a workstation. The imaging resolution of the DESI‐MSI system is ≈50 µm with a spectral resolution of 0.02 m/z. An in‐house customized NIR Raman modality with a long working distance objective as well as an interchangeable commercial probe (RPB785, InPhotonics Inc) was implemented (Figure 1a). At a 45° angle, it was focused the 785 nm laser light (B&W Tek BRM‐785‐0.55‐1000.22‐FC, 600 mW) (≈200 mW on the sample) onto the same spot as the electro‐sprayer to enable correlative Raman spectroscopy. The Raman scattered light was passed back through the collection optics and focused onto a 105 µm fiber acting as an optical pinhole before entering a high‐throughput NIR‐optimized spectrometer (Princeton Instruments Acton LS785 using a Princeton Instruments PIXIS 400BR 750–1100 nm). A digital acquisition board (National Instruments USB X SERIES Multifunction DAQ) was connected to the I/O control box of the DESI‐MS system and was used to synchronize the acquisition.

### Handheld Fibre‐Optic Raman System for Bulk Tissue Spectroscopy

The use of the same spectrometer and probe as in the integrated Raman/DESI‐MS imaging instrument ensured model transferability. We used the NIR Raman probe (RPB785, InPhotonics Inc.) to perform the bulk tissue measurements. The Raman probe was connected to a 785 nm laser (B&W Tek BRM‐785‐0.55‐1000.22‐FC, 600 mW) (≈200 mW on the sample) with a 105 µm excitation fiber and a NIR spectrometer (Princeton Instruments Acton LS785 using a Princeton Instruments PIXIS 400BR ≈750–1100 nm). Comprehensive software has been developed in the Matlab 2022a environment to provide real‐time data collection and analysis.

### Raman/DESI‐MS Imaging Alignment Protocol

To align the two modalities, an alignment protocol was created using lines of calibration ink on magnesium fluoride (MgF_2_) substrate that gives rise to intense fluorescence and DESI‐MS signal. The alignment of Raman spectroscopy and DESI‐MS in the x‐y lateral plane was iteratively optimized to achieve maximum fluorescence and MS signal. First, the DESI‐MS sprayer was focused and optimized on a magnesium fluoride (MgF_2_) slide (Global Optics Ltd) with black calibration ink. While synchronously measuring the signal intensity of the black ink on both the DESI‐MS and Raman systems (fluorescence), the Raman focusing module was optimized in the x‐y plane for maximum intensity. This was performed multiple times in both x and y directions until the focusing spots of the two systems overlapped.

### Raman/DESI‐MS Imaging of Brain Tissues

All Raman and DESI‐MS spectra were collected on the integrated Raman/DESI‐MS system simultaneously. Coronal cut mouse brain sections were imaged (≈10 000 × 12 000 µm (spatial resolution of 50 µm)) by raster scanning (Figure S2, Supporting Information) across the entire tissue surface. Each Raman and DESI‐MS spectrum was collected with an acquisition time of ≈0.5 s and laser power on the sample of 200 mW. The electro sprayer supplied MeOH at a flow rate of 2 µl min^−1^, with a nebulizing gas (nitrogen) pressure of 0.5 bar. The electro sprayer was angled at 65°, and the Raman probe at 45° to the surface of the tissue.

### Charging of MgF_2_ Substrate

Raman spectroscopy was poorly compatible with standard microscope glass slides and requires Raman‐compatible substrates such as MgF_2_ or Raman grade CaF_2_. For this reason, we developed a protocol to electrostatically charge MgF_2_ slides using poly‐l‐lysine, making Raman spectroscopy and DESI‐MS imaging fully compatible with correlative tissue imaging. This is necessary since the DESI gas pressure can destroy tissue that is not adhering to the substrate. 1% poly‐l‐lysine in H_2_O (Merck SLCL3300) was mixed with milli‐Q water in a ratio of 1/9. Cleaned MgF_2_ slides were then placed into a petri dish, and the poly‐l‐lysine solution was poured over the slides leaving ≈10 mm of the solution above the slides. The slides were then stored in the solution for 24 h at 25 °C, with a cover on top to avoid contaminants. Afterward, the slides were removed from the solution, air‐dried, and stored in a microscope slide container at room temperature.

### Healthy Mouse Brain Sample Preparation

Female Albino CD1 wildtype mice aged 6 weeks were used in the experiments in accordance with UK Home Office Procedures. Brain dislocations were carried out immediately after animal sacrifice by neck dislocation. Using sterile instruments, the skull was exposed by cutting the skin on top of the head. Occipital and interparietal bones were cut. An incision was then made in the skull along the sagittal and parietal sutures. A hole was made in the skull at the junction of frontal and parietal bones, where the tip of a scissor was inserted to crack open the calvaria. The remaining skull was removed to expose the brain completely and remove the remaining nerves and peduncles. The brain was then taken out of the skull and immediately transferred to liquid nitrogen. The brains were stored at −80 °C until sectioning. Coronal sections of the mouse brains were cryosectioned (Bright OFT Refrigerated Cryostat) with a 40 µm thickness and thaw mounted on charged MgF_2_ slides. The samples were stored at −80 °C until analysis. Prior to imaging, the tissues were thawed at room temperature.

### Mouse Model of Transient Cerebral Ischemia‐Reperfusion Injury

Male C57BL/6J mice, aged 10–12 weeks and maintained on a 12‐h light cycle from 0700 to 1900, were used for experiments in accordance with UK Home Office Procedures. Mice were pre‐treated i.p. with 5 mg k^−1^g of methadone prior to the onset of surgery and induced to a surgical plane of anesthesia with inhalation of 4% isoflurane in 30% oxygen, reduced to 1.0–1.5% isoflurane for maintenance. The middle cerebral artery was transiently occluded for 60 min, using a modification of the Longa technique,^[^ ^26^ ^]^ followed by recovery from anesthesia and reperfusion for 72 h with analgesia provided. Brains were washed in heparin/saline and directly flash frozen. Brains were cryosectioned with three sections (40 µm) cut near −2 mm relative to bregma, placed on electrostatically charged MgF_2_ slides, and stored at −80 °C until imaging.

### MS/MS

Utilizing tandem mass spectrometry (MS/MS) fragmentation, species identification within mouse brain specimens was conducted via MS/MS. The electrospray ionization voltage was fixed at 4.5 kV, for the acquisition of negatively charged ions. Methanol was delivered at a flow rate of 5 µL min^−1^ and subsequently atomized through the application of nitrogen gas at a pressure of 5 bar. To determine the identities of MS/MS fragment ions, the fragmentation patterns of chosen mass spectrometry peaks were compared with those available in online MS/MS spectral repositories, specifically MassBank and Lipid Maps. All MS/MS spectra were identified using MassBank, however, ST 24:1 had no recorded MS/MS spectra and was identified using 888.6249 m/z value in Lipid Maps and previous studies on DESI‐MS of brain tissues. Cer 36:1;O_2_ was identified using 564.5 m/z ([M−H]^−^) as the precursor ion, due to difficulty of fragmenting the 600.5 m/z ([M+Cl]^−^).

### Raman Spectral Preprocessing

The raw Raman spectra were first background subtracted. For each tissue section, a region of 10 × 10 pixels outside the tissue area was empirically chosen, and the mean spectra of the region were used as a background spectrum for each tissue sample, respectively. The spectra were then calibrated to reduce etalon artifacts (Figure S8, Supporting Information): The mean spectrum using an average of multiple reference measurements (10 spectra) was fitted with a 5th‐order polynomial fit and subsequently divided with a polynomial fit of green glass fluorescence. This calibration was then multiplied onto all the raw spectra to suppress etalon artefacts.^[^ The Raman processing adhered to standard methods including autofluorescence removal, smoothing, and normalization. In this work, we found that a 2nd‐order constrained polynomial fitted to the spectra efficiently could remove the autofluorescence background. Each Raman spectrum was smoothed using a Savitzky–Golay filter (zeroth order, window: 3) and finally normalized to the integrated area to ensure comparability between images.

### DESI‐MS Spectral Preprocessing

The 1000 most prominent peaks of the DESI‐MS images were exported to a text file using HDI software (HDI version 1.5) and imported into Matlab 2022a. In Matlab, the DESI‐MS peaks were sorted using the function *mspeaks* (conversion of MS data to a peak list)*, mspalign* (alignment of MS spectra in peak list to a selected range reference peaks)*, msppresample* (resample aligned MS spectra while preserving selected peaks)] using the Matlab Bioinformatics toolbox. The list of spectra was up‐sampled to create a uniform axis (to ensure a resolution of 0.02 m/z). The up‐sampled MS spectra were then peak‐aligned using the Matlab function *mspalign*. After peak alignment, to reduce the memory size for computations, the spectra were down‐sampled by removing zeros.

### Image Masking and Background Removal

A simple mask was created for each tissue section to remove irrelevant background data (e.g., pixels from the MgF_2_ substrate) by taking the spectral sum of each pixel and removing any pixels under a lower threshold determined empirically for each image. This efficiently removed the substrate background.

### Co‐Registration

While acquired on a unified Raman and DESI‐MSI system the preprocessed Raman and DESI‐MS images were co‐registered with a feature selection approach using functions (*cpselect*) from the Matlab Image Processing Toolbox.

### Heterospectral Correlation Analysis

Heterospectral 2D correlation analysis (2D‐COS)^[^ ^13e^ ^]^ was performed on the preprocessed Raman and DESI‐MS data. The synchronous correlation spectra were used to investigate the relationship between Raman and DESI‐MS peaks.

### Partial Least Squares (PLS) Regression Modeling

Partial least squares (PLS) regression models were iteratively trained for each m/z peak in the reduced DESI‐MS spectra. The spectral data was split into a training and independent test data set (i.e., independent mice brain). PLS regression of the Raman spectra was performed against the individual DESI‐MS spectral peaks in the training data set. Mean centering was performed to reduce model complexity. An optimized number of latent variables (LVs) determined by leave‐one‐section‐out cross‐validation of the training set were used to minimize the root mean square error of cross‐validation (RMSECV). The independent test data set was used to validate the regression model by predicting the DESI‐MS spectra from the corresponding Raman spectra. To quantify the prediction capabilities for each m/z abundance, the image quality metrics, PSNR and SSIM, as well as MSE were calculated. All computation in this work was performed on a workstation (AMD Ryzen) with 64 GB of memory.

### Statistical Analysis

Multiple comparisons were calculated using one‐way analysis of variance (ANOVA) using a post hoc Tukey test) in Origin Pro 2022b.

## Conflict of Interest

TV is co‐founder and shareholder of Hypervision Surgical Ltd. The remaining authors report no further conflicts of interest.

## Author Contributions

M.J. performed the experiments, interpreted the data, generated the figures, and wrote the manuscript. S.L. performed experiments and contributed to scientific discussion and data analysis. E.S. and D.M. performed experimental work and contributed to scientific discussion. A.A.B., K.F.D., S.J.C., and G.E.M. contributed to scientific discussion, animal experiments, and data interpretation. K.L.A.C., M.P., and C.C. contributed to the scientific discussion and data interpretation. T.V. contributed to scientific discussion, data interpretation, and data analysis. V.A. contributed with scientific discussion, instrument development, and MS data interpretation. M.S.B. designed the study, developed the instrument, interpreted the data, and wrote the manuscript.

## Supporting information

Supporting Information

## Data Availability

The data that support the findings of this study are available from the corresponding author upon reasonable request.
