# Supplementary material for: Opto‐Lipidomics of Tissues
Source: Adv Sci (Weinh). 2023 Dec 25;11(14):2302962. doi: 10.1002/advs.202302962 (PMC11005704; doi:10.1002/advs.202302962)
Supplement: Supplementary file 1 — Supporting Information [file ADVS-11-2302962-s001.pdf]

ADVANCED SCIENCE
Open Access

Supporting Information

for *Adv. Sci.*, DOI 10.1002/advs.202302962

Opto-Lipidomics of Tissues

*Magnus Jensen, Shiyue Liu, Elzbieta Stepula, Davide Martella, Anahid A. Birjandi, Keith Farrell-Dillon, Ka Lung Andrew Chan, Maddy Parsons, Ciro Chiappini, Sarah J. Chapple, Giovanni E. Mann, Tom Vercauteren, Vincenzo Abbate and Mads S. Bergholt**

Supporting information

Opto-lipidomics of tissues

Magnus Jensen[1], Shiyue Liu[1,2], Elzbieta Stepula[1], Davide Martella[1], Anahid A. Birjandi[1], Keith Farrell-Dillon[3], Ka Lung Andrew Chan[2], Maddy Parsons[4], Ciro Chiappini[1], Sarah J. Chapple[3], Giovanni E. Mann[3], Tom Vercauteren[5], Vincenzo Abbate[6], Mads S. Bergholt[1*]

[1]Centre for Craniofacial and Regenerative Biology, King's College London, London SE1 9RT, UK

[2]Institute of Pharmaceutical Science, King's College London, SE1 9NH, UK.

[3]King's British Heart Foundation Centre of Research Excellence, School of Cardiovascular and Metabolic Medicine & Sciences, Faculty of Life Sciences & Medicine, King's College London, 150 Stamford Street, London SE1 9NH, UK

[4]Randall Centre for Cell and Molecular Biophysics, King’s College London, London, SE1 1UL, UK

[5]School of Biomedical Engineering and Imaging Sciences, King's College London, WC2R 2LS, UK

[6]Department of Analytical, Environmental and Forensic Sciences, King's College London, 150 Stamford Street, London SE1 9NH, UK

Supplementary Figure S1

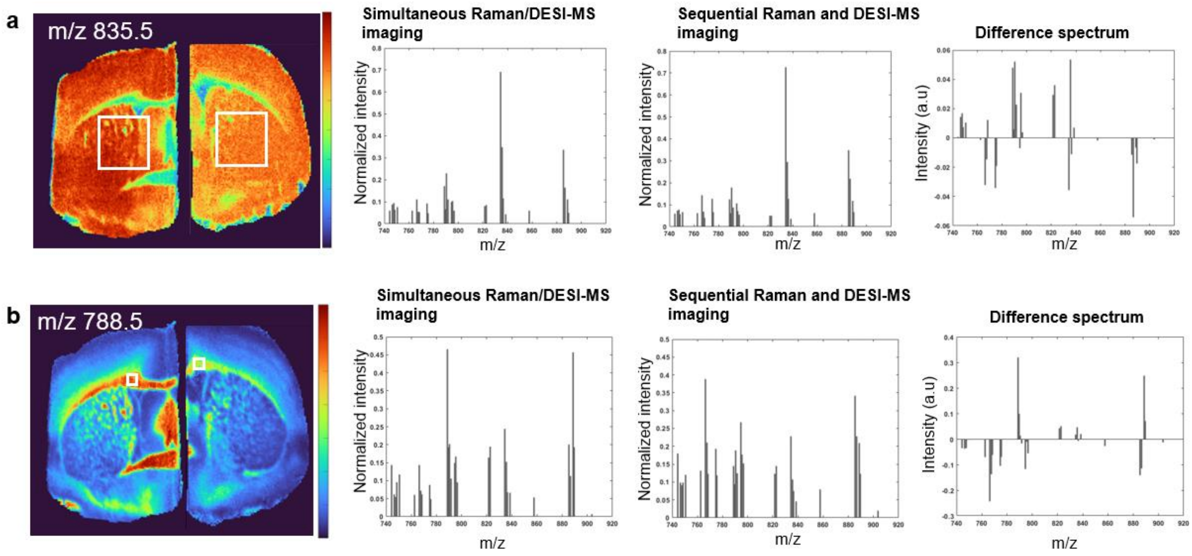

Supplementary Figure S1. A 40 µm thick section from a fresh-frozen mouse cerebrum was cut it into two halves by a surgical scalpel. Both halves were stored in a -80°C freezer until imaging. The left hemisphere remained frozen and underwent imaging using a Raman/DESI-MS system, totaling ~4 hours. Conversely, the right hemisphere underwent standard Raman spectroscopy (Renishaw Raman microscopy, 785 nm, 50X objective) followed by DESI-MS imaging, with a total imaging time of approximately ~4 hours + ~4 hours. The DESI-MS spectra were normalized to the total ion count for investigating changes in relative abundances. This experimental setup provided an internal control within the same tissue, enabling the assessment of whether the 785 nm focused laser excitation exposure over the course of ~4 hours in ambient conditions had any noticeable impact on the quality of DESI-MS imaging. a) DESI-MS image of m/z 835.5 associated with grey matter reveals a relative change in abundance after sequential imaging with Raman and DESI-MS. b) A DESI-MS image of m/z 788.5 associated with white matter displays a relative change in abundance across the corpus callosum after sequential imaging with Raman and DESI-MS.

Supplementary Figure S2

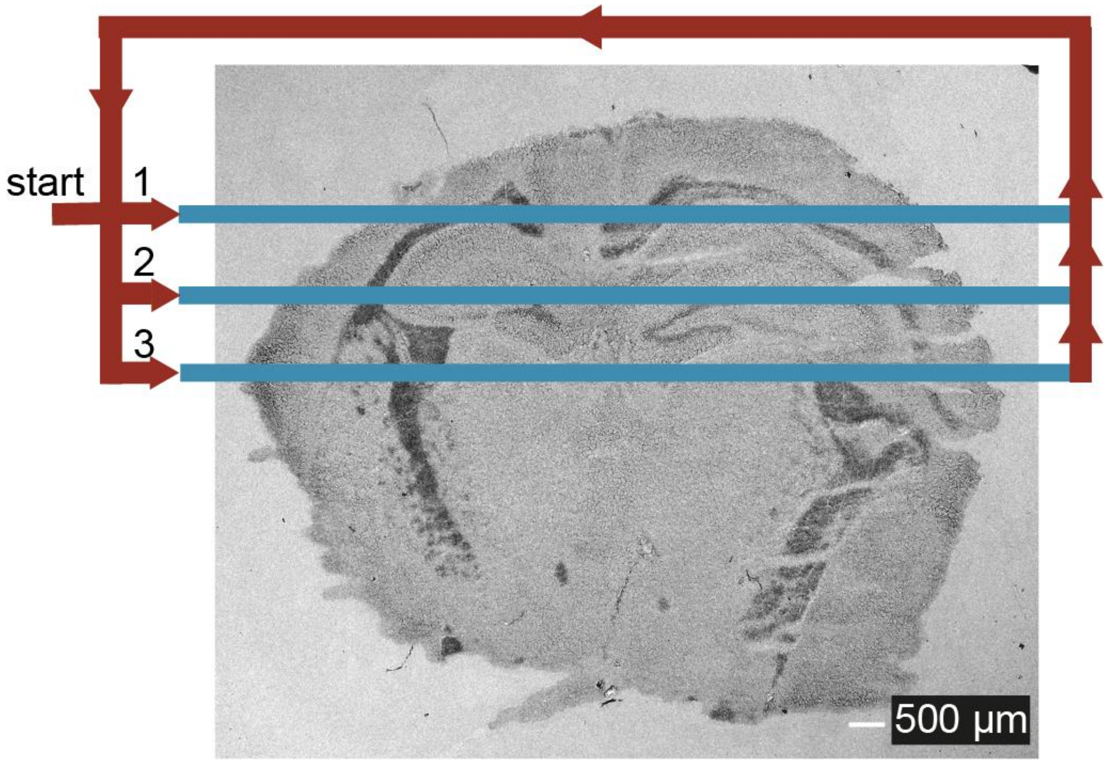

Supplementary Figure S2. The integrated Raman and DESI-MS system employs a specific scanning pattern. This pattern ensures that the Raman and DESI-MS spectra are collected exclusively during tissue sampling, as the DESI sprayer continuously supplies methanol (MeOH). The acquisition process halts when the motorized stage follows a rectangular trajectory around the sample, preventing consecutive exposure to MeOH. Also shown is the comparison of an integrated DESI/Raman imaging approach with approximately matched resolution in comparison with a sequential DESI/Raman imaging previously reported.

Supplementary Figure S3

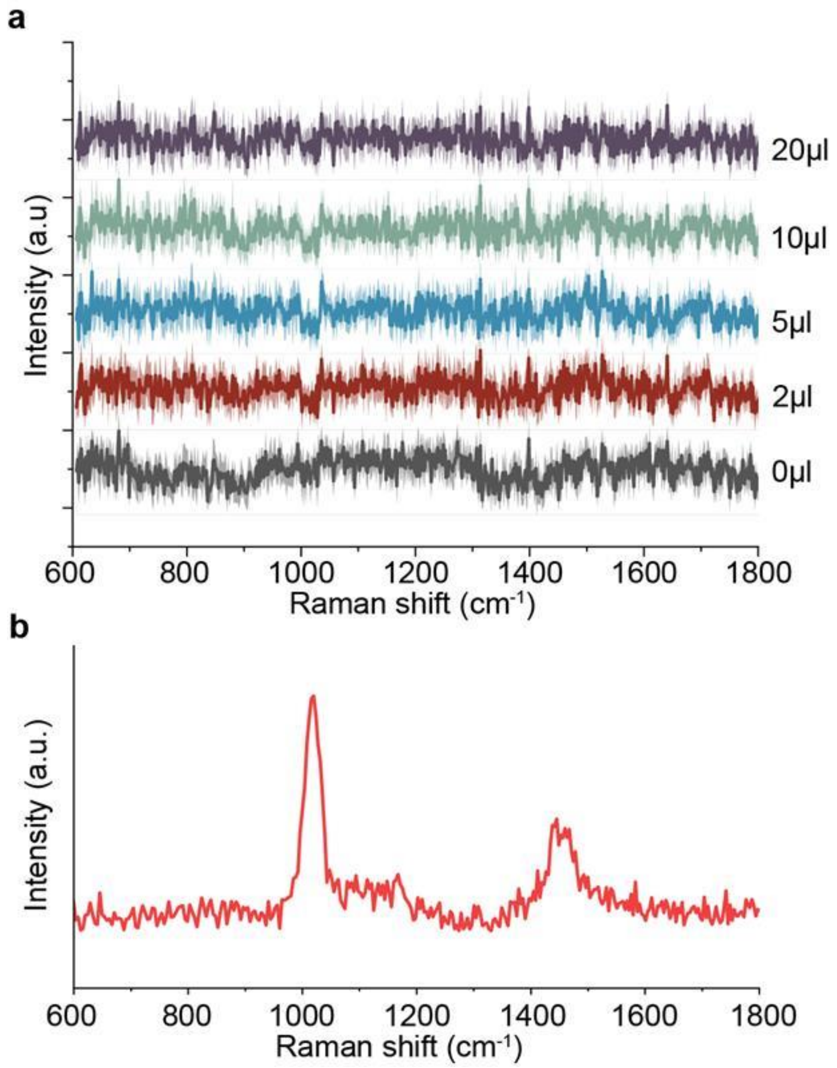

Supplementary Figure S3. a) Raman spectra ± 1 standard deviation of MgF_2 slides with varying methanol flow rates (0-20 µl/min) were collected to investigate the potential contamination of the Raman signal by MeOH peaks. A total of 5 spectra were collected at each flow rate. The integration time for each spectrum was 0.5 seconds. The Raman spectra show no significant contamination by MeOH peaks, even at the highest flow rate of 20 µl/min. b) Pure MeOH Raman spectrum showing distinct peaks at near ~1000 cm^{-1} and ~1450 cm^{-1}.

Supplementary Figure S4

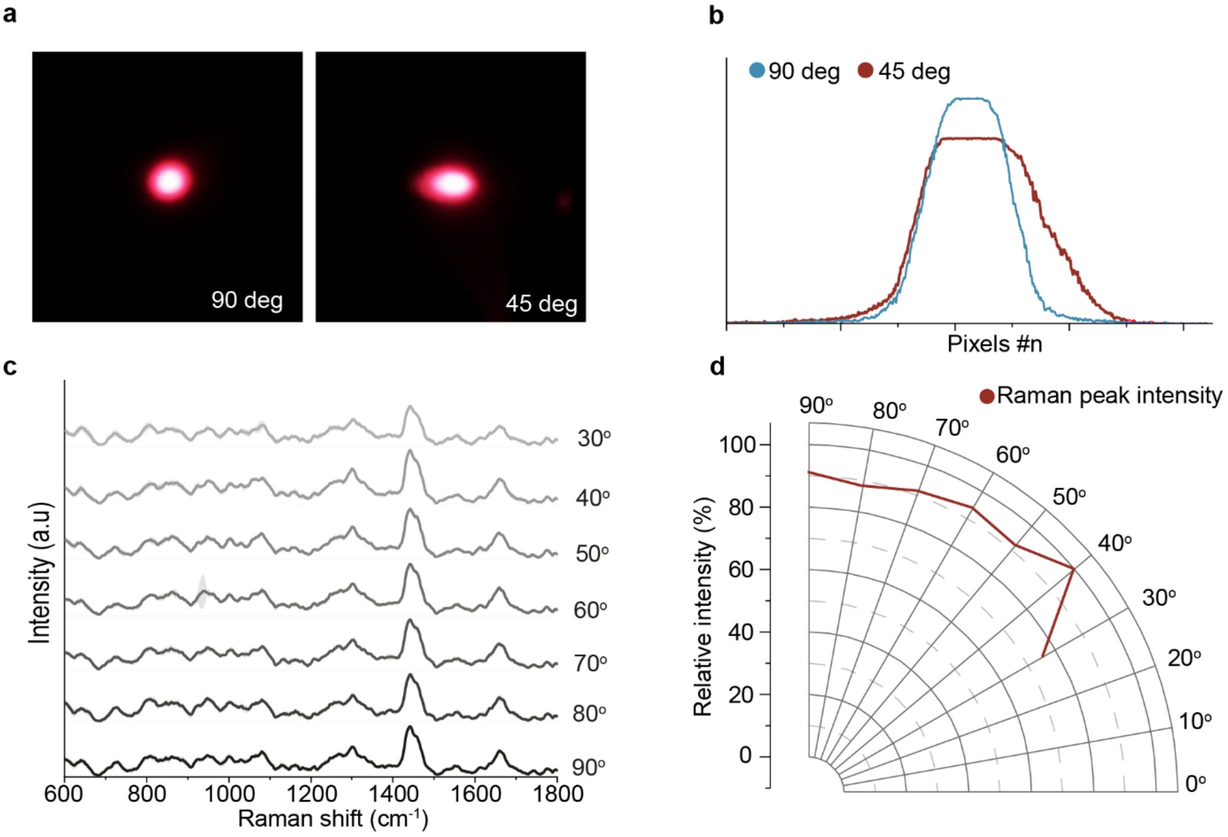

Supplementary Figure S4. Since the integrated Raman modality collects spectra at an incident angle relative to the surface, we characterised the angular dependence of the tissue Raman spectrum intensity. a) Spot shape for the 785 nm laser excitation at 90 degrees and 45 degrees incident angle. b) Laser excitation distribution at 90 degrees and 45 degrees incident angle. c) Raman spectra of brain tissue at various incident angles 30 - 90 degrees. d) Polar plot of the relative intensity of the 1445 cm^{-1} peak at various incident angles 30 - 90 degrees. The tissue Raman signal shows a subtle increase in intensity (~10%) between the normal to the surface and 40 degrees (1445 cm^{-1} peak, $p<0.05$, one-way ANOVA), which is expected for a well-focused spot on a thin tissue section.

Supplementary Figure S5

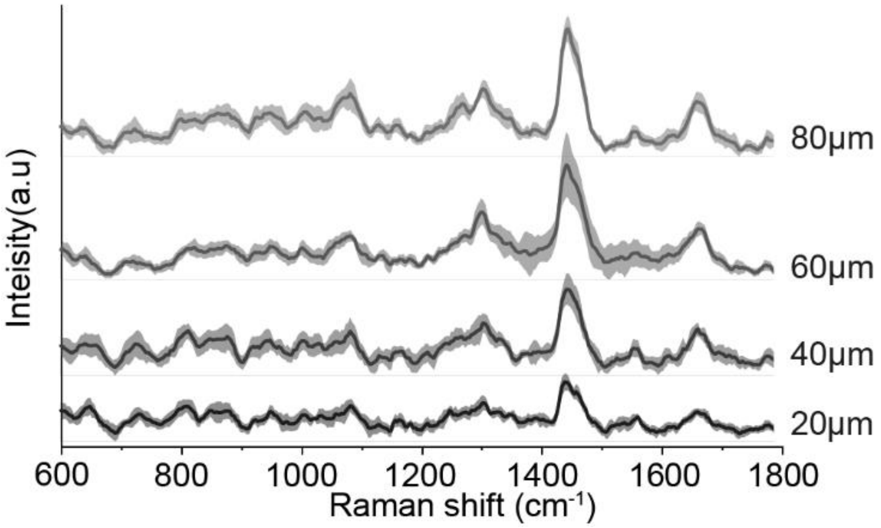

Supplementary Figure S5. The effect of tissue thickness (20, 40, 60, and 80 µm) on the Raman signal intensity on healthy mouse brain tissue revealed that 40 µm tissue sections provide a good compromise between signal intensity and tissue thickness corresponding to ~3x of a typical cell size.

Supplementary Figure S6

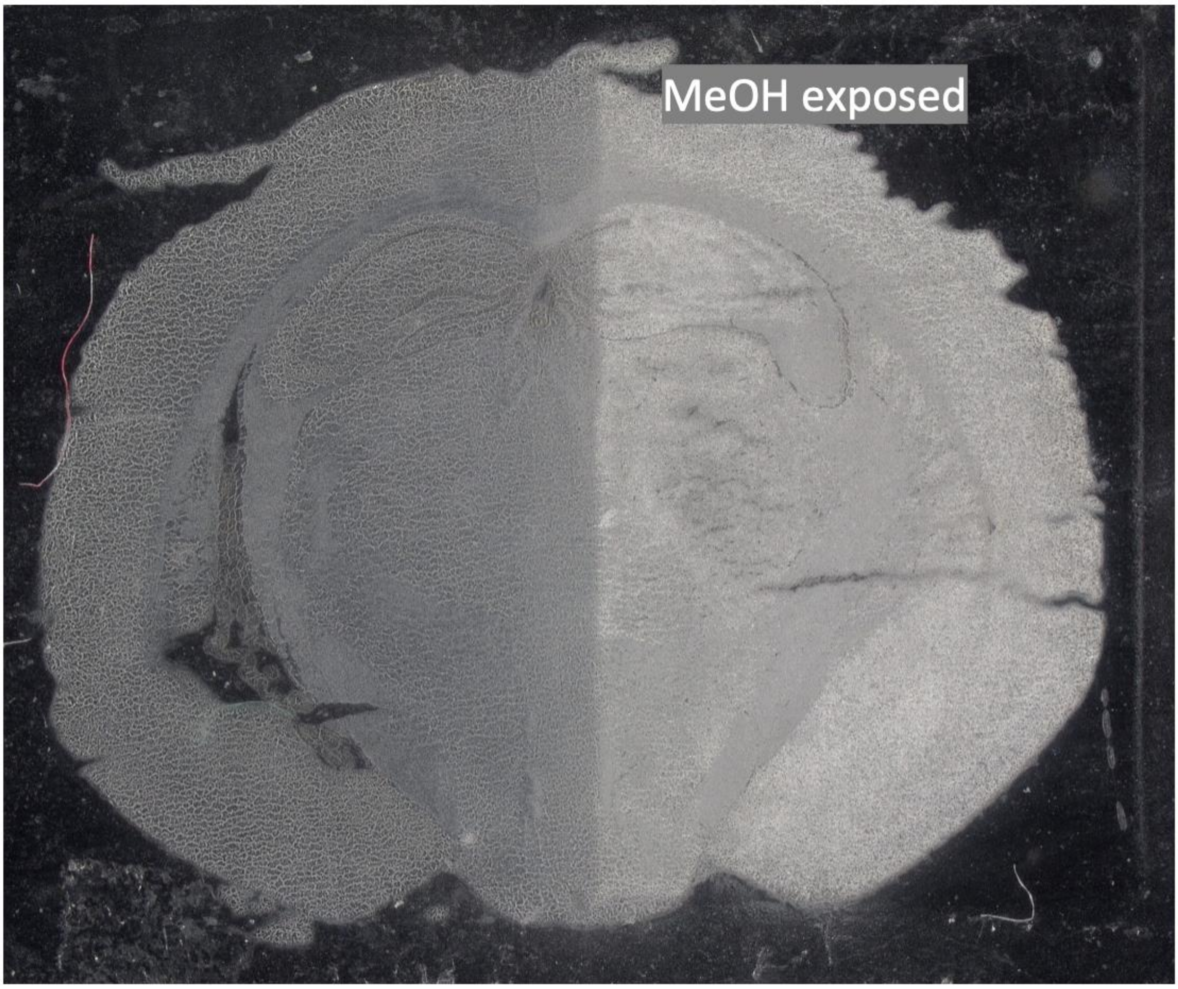

Supplementary Figure S6. Brightfield image of a mouse brain section demonstrates the effect of DESI-MS measurements of the tissue. The portion on the right side of the section was exposed to MeOH. Upon visual inspection, it is evident that the tissue sample is effected by DESI-MS imaging.

Supplementary Figure S7

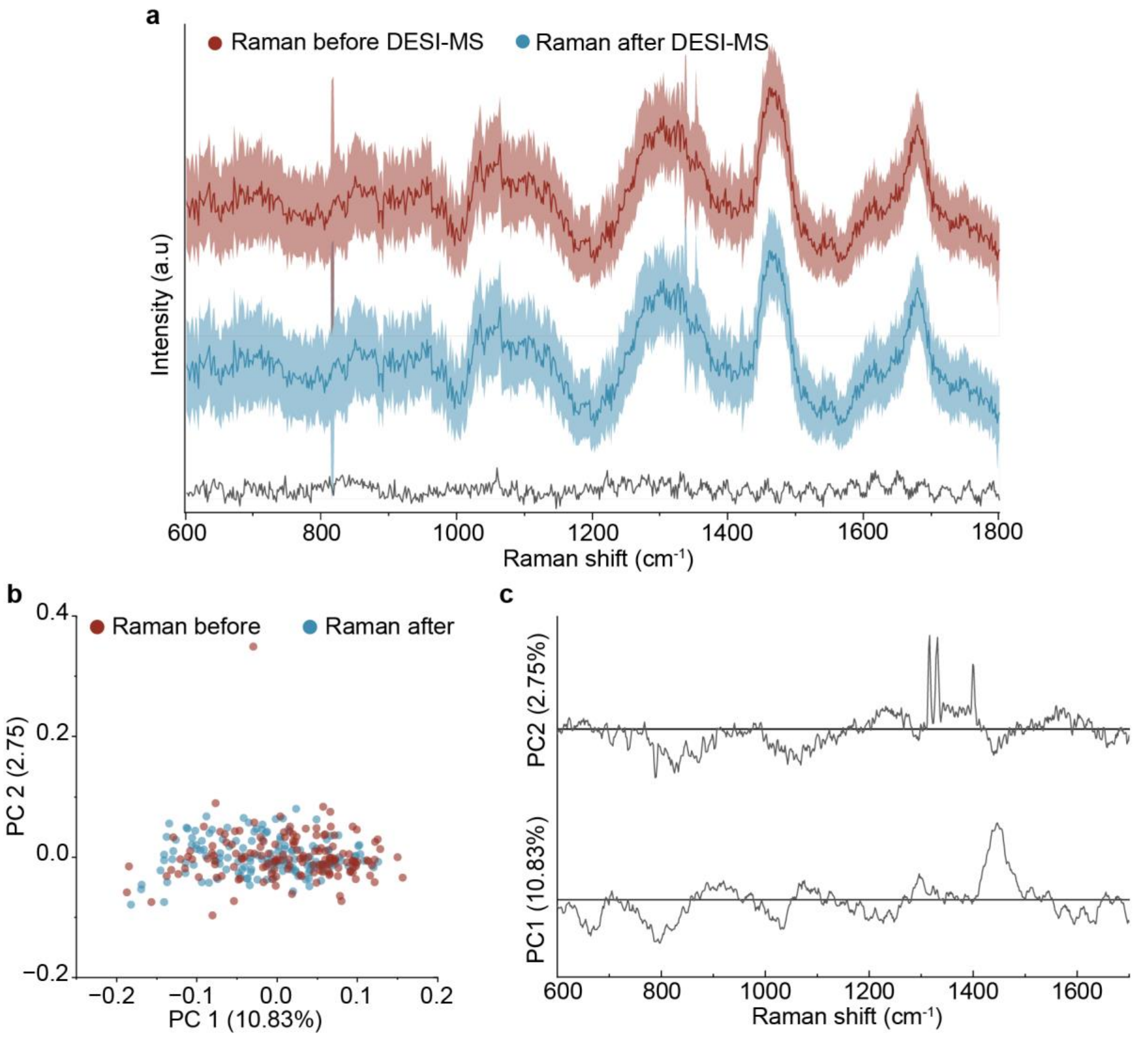

Supplementary Figure S7. a) Mean Raman spectra of mouse brain tissue before DESI-MS measurement (red) and after DESI-MS measurement (blue) (0.5 sec integration time). Also shown is the difference residual spectrum (black). b) Principal component analysis (PCA) scores on the Raman data (n=150 before DESI-MS, n=150 after DESI-MS). c) PCA loadings reveal noise sources (e.g., sutble etaloning effects) that were more prominent than any chemical changes induced by DESI.

Supplementary Figure S8

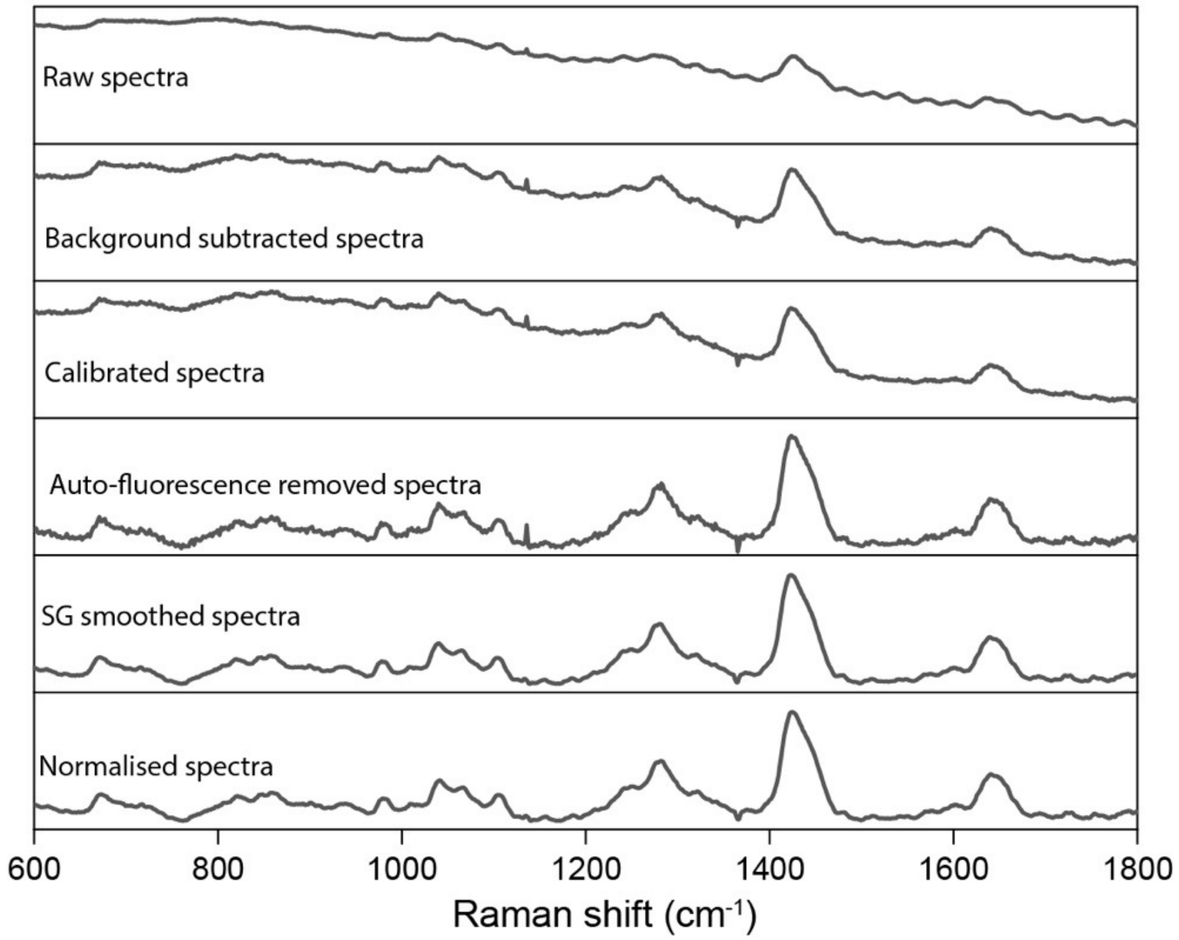

Supplementary Figure S8. Sequential preprocessing of Raman spectroscopy, including calibration for etaloning effects, background subtraction, autofluorescence removal, Savitzky Golay (SG) smoothing and normalisation.

Supplementary Figure S9

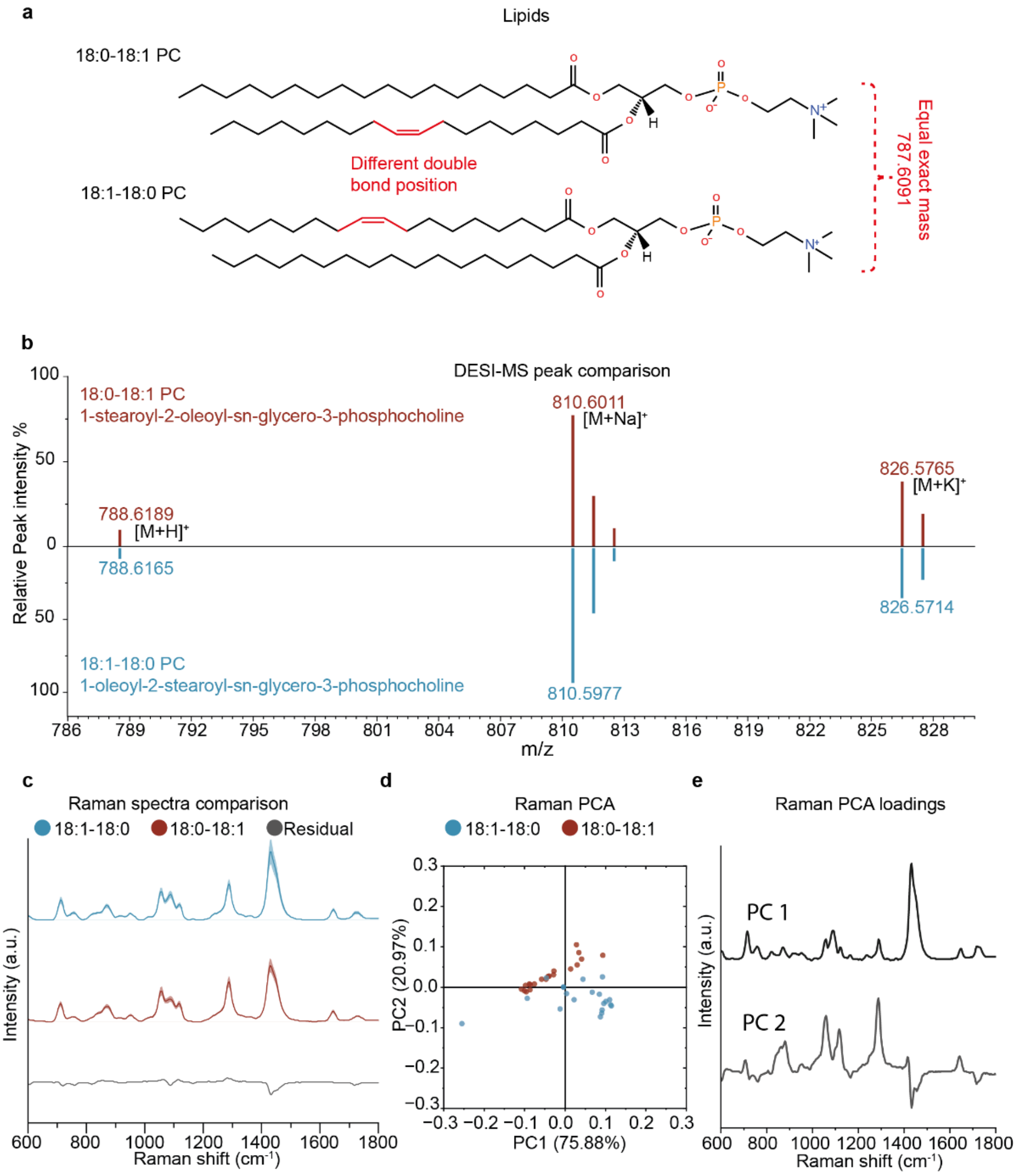

Supplementary Figure S9. a) PC18:1-18:0(1-oleoyl-2-stearoyl-sn-glycero-3-phosphocholine) and PC18:0-18:1(1-stearoyl-2-oleoyl-sn-glycero-3-phosphocholine) of identical elemental composition but with a double band located in a different position of the alkyl chains. b), DESI-MS spectrum of the two lipids (mirrored around zero for clarity) showing consistent overlap of the MS peaks. These lipids cannot be discriminated using DESI-MS alone. c) Average Raman spectra ±1 standard deviation (SD) of the two lipids. Also shown is the difference spectrum (18:1-18:0 minus 18:0-18:1). d) Principal component analysis (PCA) scores showing complete separation of the two lipids. e). PCA loadings revealing subtle peak changes and shifts in both PC1 and PC2.

We established the complementarity between optical spectroscopy and DESI-MS. Species bearing identical elemental composition, such as structural isomers, cannot be differentiated using DESI-MS alone. While tandem MS/MS can often discriminate isomers, the use of Raman spectroscopy could offer a rapid and highly efficient alternative. We investigated if Raman spectroscopy can be used to resolve subtle molecular identity by measuring two very similar lipids: PC18:1-18:0(1-oleoyl-2-stearoyl-sn-glycero-3-phosphocholine) and PC18:0-18:1(1-stearoyl-2-oleoyl-sn-glycero-3-phosphocholine) of equal molecular mass but with an unsaturation positioned differently (Figure S9a). Positive ion mode DESI-MS spectra showed a complete overlap of the m/z peaks corresponding to fragments the two lipids (Figure S9b). The average Raman spectra ± 1 SD of the two lipids (Figure S9c) showed well-known peaks associated with phospholipids, e.g., 1450 cm^{-1} (CH_2 deformations), 1650 cm^{-1} υ(C=C) and 1745 cm^{-1} υ(C=O). We also calculated the difference Raman spectrum ± 1 SD revealing subtle differences with consistent spectral peak shifts, particularly at 1066 cm^{-1} and 1426 cm^{-1} corresponding to υ(C-C) and β(CH_2) bonds of the lipids (Figure S9c). Two-component PCA analysis and linear discriminant analysis provided clear discrimination between the two lipid molecules (Figure S9d-e). This demonstrates that while DESI-MS can offer specific identification of the lipid sub-class, Raman spectroscopy can probe the subtle vibrational differences reflecting the molecular structure and resolve highly similar lipid species. Whilst this experiment was performed with purified lipids and not tissues, it provides an important demonstration of the complementarity of these two techniques. Extracting this information from complex tissues will require more comprehensive correlation with LC-MS/MS.

Purified lipid sample preparation and measurements of two nearly identical lipids: Two lipids (18:1-18:0 PC, 18:0-18:1 PC) (Merck) and stored at -20°C until sample preparation. A ratio of 2 mg/ml lipids to chloroform solution was prepared for each different lipid. Multiple droplets of the solutions were deposited separately on CaF_2 slide discs. After air-drying for 10 minutes, a thin lipid film formed on the slide. The slides were then analysed on the DESI-MS system, in positive ion mode. For the Raman analysis, 5 mg of the dry lipids were placed on an MgF_2 slide and measured using 785 nm laser excitation. The excitation laser power for Raman imaging was 74 mW under 50X dry objective lens with 1 second integration time. We report the average of 20 spectra sampled at random locations for each lipid.

Supplementary Figure S10

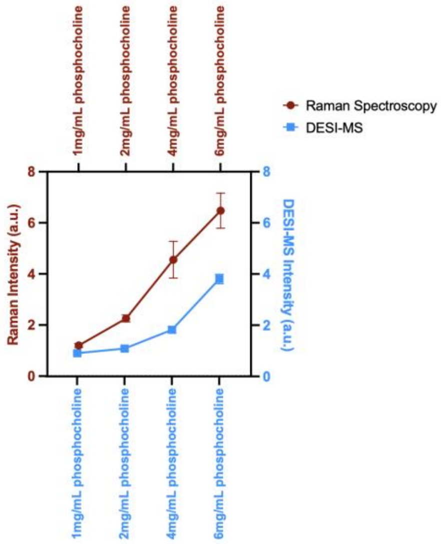

Supplementary Figure S10. Relative Raman spectroscopic and DESI-MS quantification of PC18:1-18:0(1-oleoyl-2-stearoyl-sn-glycero-3-phosphocholine) relative to L-α-Phosphatidylcholine (Sigma P5394). The lipid mixtures were analysed under Raman microscope and DESI-MS. We recorded 10 Raman spectra at random locations for each lipid mixture and used non-negative least square method to provide relative quantification of the PC18:1-18:0 lipid. We also recorded 49 DESI-MS spectra for each lipid mixture film and analysed the PC18:1-18:0 concentration by normalising to L-α-Phosphatidylcholine. For DESI-MS there is non-linear behaviour proportional to the concentration likely due to the absence of stable–isotope internal standard.

Relative quantification of two lipids in controlled experiment: Two lipids, PC18:1-18:0(1-oleoyl-2-stearoyl-sn-glycero-3-phosphocholine) and L-α-Phosphatidylcholine (Sigma P5394) were stored at -20 ˚C until sample preparation. We prepared 8 mg/mL PC18:1-18:0 stock solution and 40 mg/mL L-α-Phosphatidylcholine stock solution with chloroform. Then different concentration PC18:1-18:0 solutions were mixed with Phosphatidylcholine solutions and final concentration of 1 mg/mL, 2 mg/mL, 4 mg/mL and 6 mg/mL PC18:1-18:0 solutions were achieved. Each PC18:1-18:0 solution mixture had 10 mg/mL L-α-Phosphatidylcholine serving as a reference. We deposited multiple 5 μL droplets on CaF_2 slide from each solution mixture separately. After air-drying for 10 minutes, thin lipid films formed on the slide. The lipid mixtures were analysed under Raman microscope and DESI-MS. We used 785 nm laser for Raman imaging and the excitation laser power was 74 mW using 50x dry objective with 1 second integration time. We recorded 10 spectra at random locations for each lipid mixture and used non-negative least square method to provide relative quantification of PC18:1-18:0 lipid. For DESI-MS imaging, we recorded 49 spectra for each lipid mixture film and analysed the PC18:1-18:0 concentration by normalising to L-α-Phosphatidylcholine.

Supplementary Figure S11

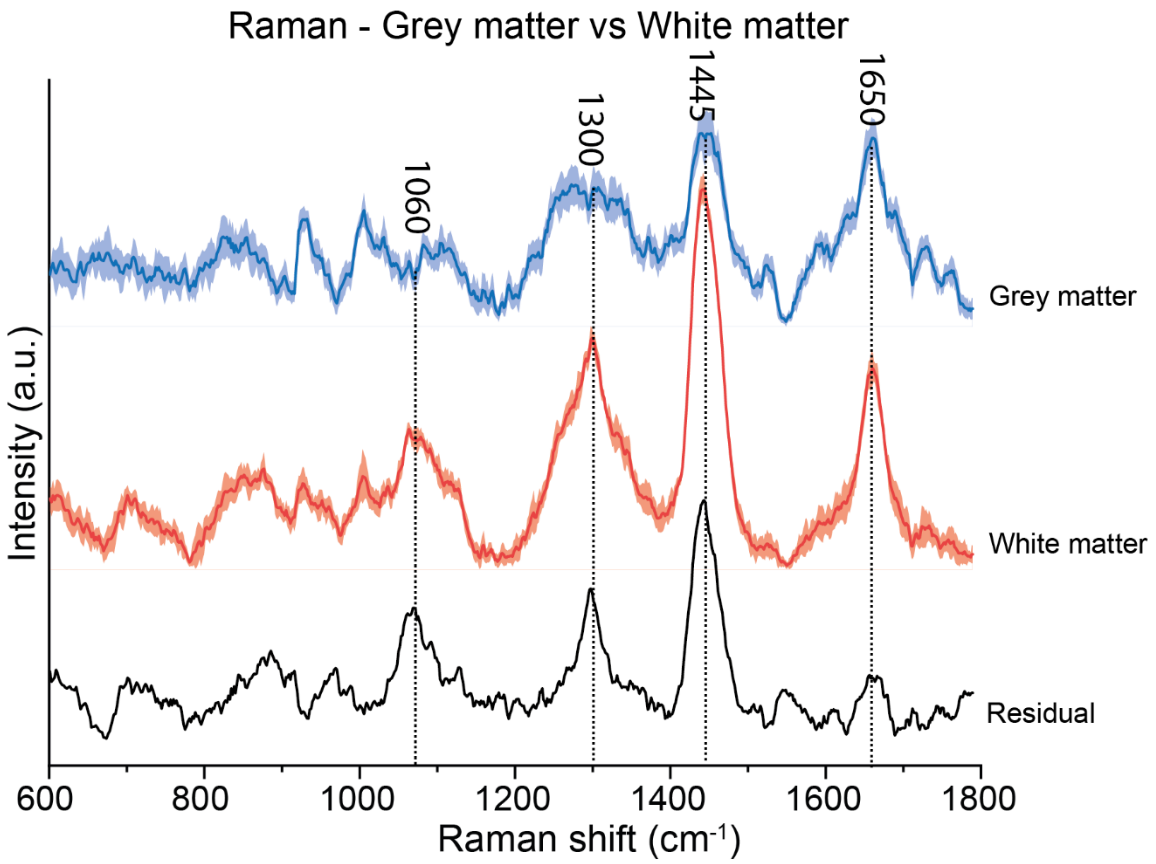

Supplementary Figure S11. Raman spectra ±1 standard deviation (SD) of grey and white matter of a mouse brain. Also shown is the residual spectrum (white matter – grey matter).

Supplementary Figure S12

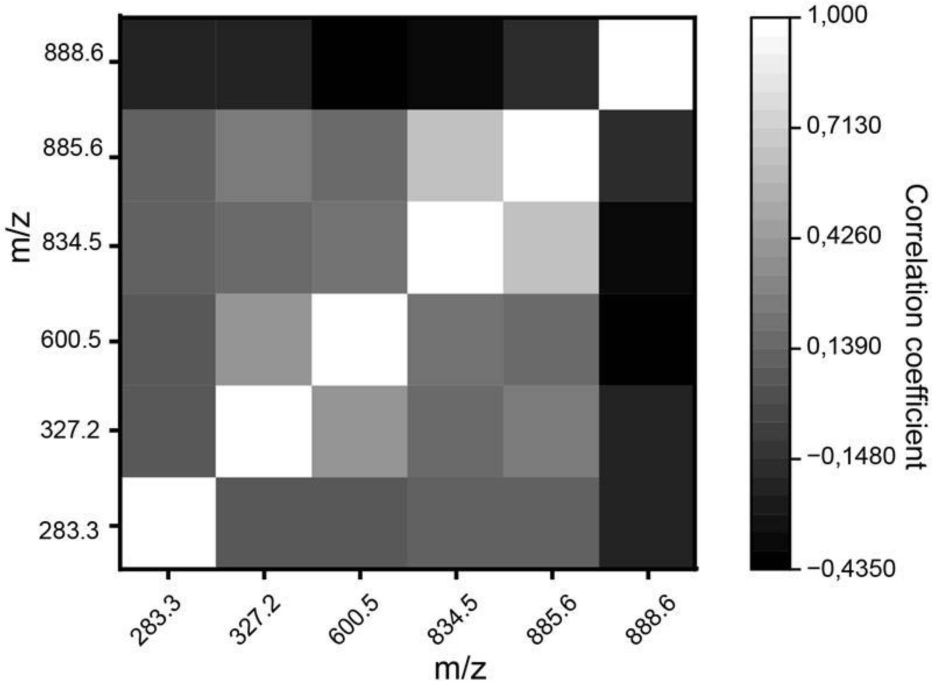

Supplementary Figure S12. Correlation coefficients of some of the major DESI-MS peaks in a mouse brain indicating that these largely show independent distributions across the tissue.

Supplementary Figure S13

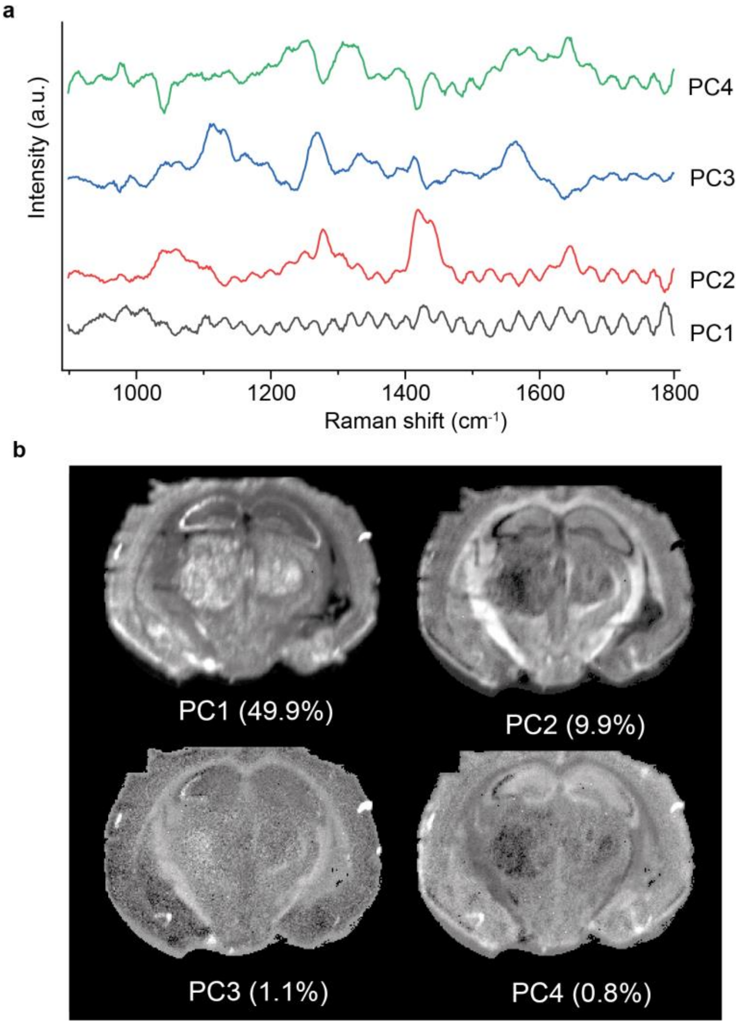

Supplementary Figure S13. a) Principal component analysis (PCA) loading of the Raman spectra of brain tissues reveals little or no specific molecular information about the tissue composition. b) PCA scores (PC1:49.9%, PC2:9.9%, PC3:1.1% and PC4:0.8%). Notice PC1 largely contain etaloning effects while the remaining PCs account for the molecular differences in the tissue.

Supplementary Figure S14

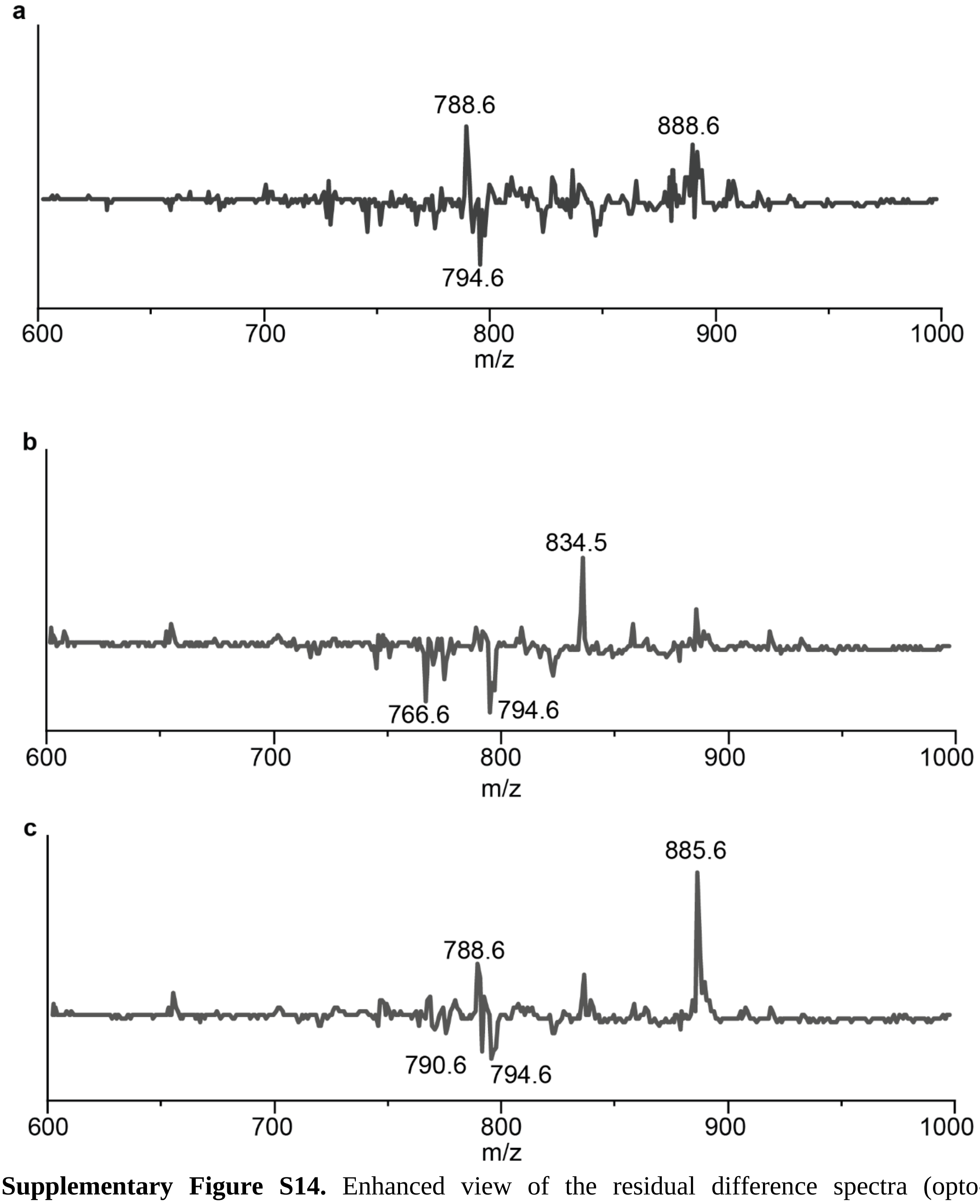

Supplementary Figure S14. Enhanced view of the residual difference spectra (opto-lipidomics – DESI-MS) adapted from Figure 3c.

Supplementary Figure S15

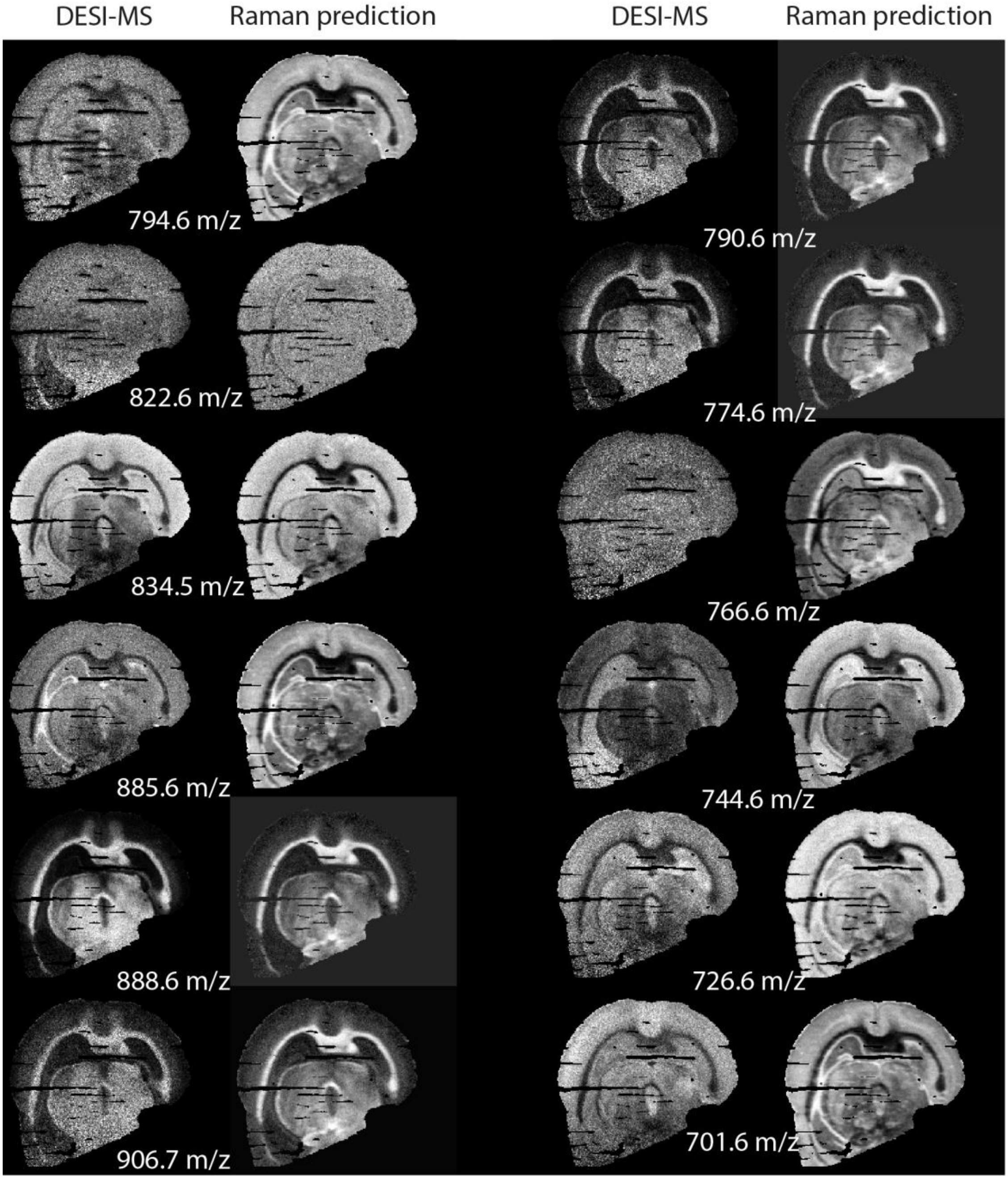

Supplementary Figure S15. The 12 most intense m/z peak abundance images of DESI-MS. Also shown are the Raman predicted (opto-lipidomics).

Supplementary Figure S16

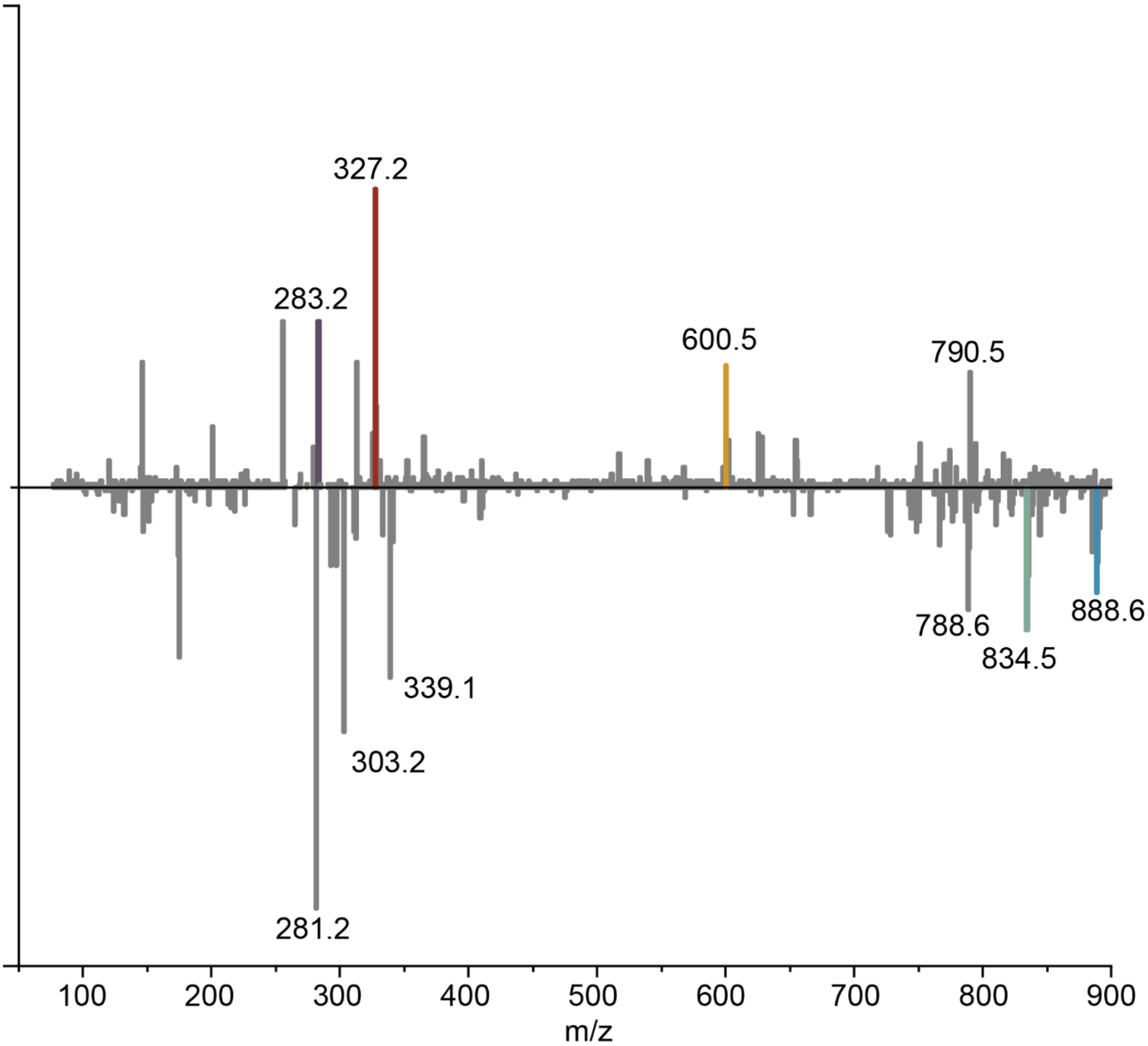

Supplementary Figure S16. Enhanced view of the residual difference spectra (opto-lipidomics – DESI-MS) adapted from Figure 4c.

Supplementary Figure S17

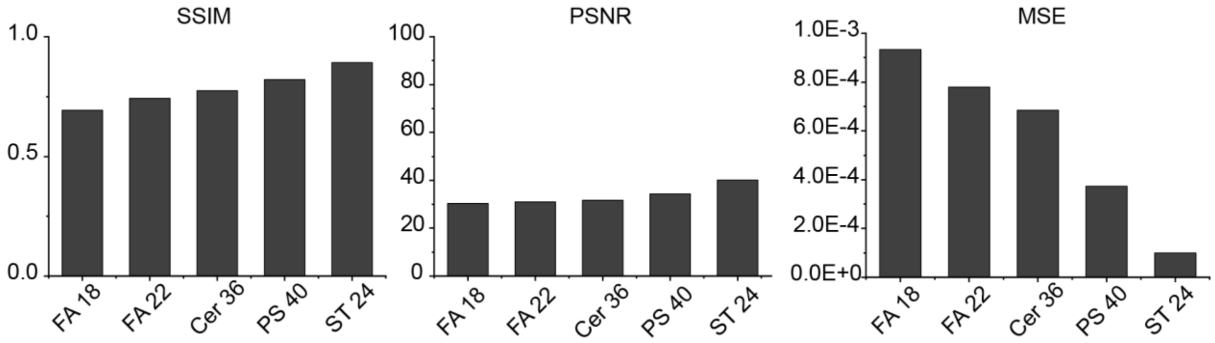

Supplementary Figure S17. Image quality metrics: a) Structural similarity index (SSIM), b) Mean squared error (MSE) and c) Peak signal to noise ratio (PSNR), for FA 18:0, FA 22:6, Cer 36:1;O2, PS 40:6, and ST 24:1

Supplementary Figure S18

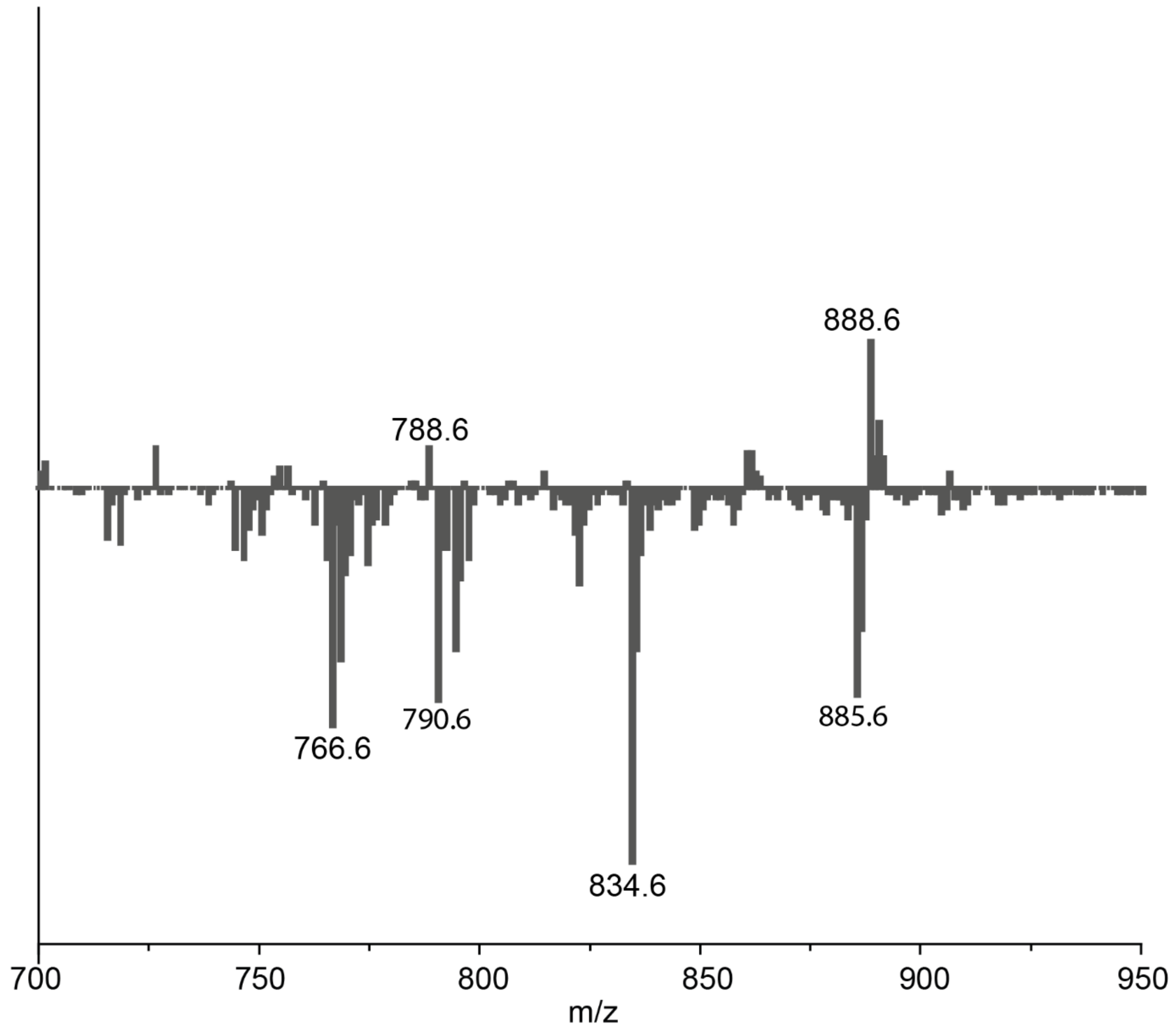

Supplementary Figure S18. Enhanced view of the residual difference spectra (opto-lipidomics – DESI-MS) adapted from Figure 5d.

Table S1

m/z acc	m/z exact	Error (ppm)	Assignment	Elemental formula	Formation
283.2653	283.2643	3.53	FA 18:0 (Stearic acid)	$C_{18}H_{35}O_2$	$[M-H]^-$
327.2338	327.2330	2.44	FA 22:6 (DHA)	$C_{22}H_{31}O_2$	$[M-H]^-$
600.5120	600.5128	-1.33	Cer 36:1;O2	$C_{36}H_{71}ClNO_3$	$[M+Cl]^-$
788.5416	788.5447	-3.93	PS 36:1	$C_{42}H_{79}NO_{10}P$	$[M-H]^-$
834.5290	834.5291	-0.11	PS 40:6	$C_{46}H_{77}NO_{10}P$	$[M-H]^-$
885.5504	885.5499	0.56	PI 38:4	$C_{47}H_{82}O_{13}P$	$[M-H]^-$
888.6249	888.6240	1.01	ST 24:1 (Sulfatide)	$C_{48}H_{90}NO_{11}S$	$[M-H]^-$

Table S1 | Table of peaks present in mouse brain experiments. m/z acc represents the observed m/z peak value. m/z exact represents the exact mass of the peak. Error represents the m/z error between the observed and exact m/z in ppm. Assignment represents the assigned species to the peak. Also shown is the elemental formula of the assigned species.

Table S2

**Precursor ion**	**Assignment**	**MassBank reported fragments**	**Observed fragments**	**MassBank Identifier**
**283.2**	FA 18:0 (Stearic acid)	265.3000, 283.3000	265.2551, 283.2654	MSBNK-Metabolon-MT000015
**327.3**	FA 22:6 (DHA)	229.1980, 283.2434, 327.2336	229.2319, 283.2846, 327.2322	MSBNK-BGC_Munich-RP029612
**564.5**	Cer 36:1;O2	237.2200, 265.2510 282.2790, 308.2940, 564.5320	237.2222, 265.2541, 283.2659, 308.2965, 564.5387	MSBNK-RIKEN_IMS-LQB00032
**788.5**	PS 36:1	152.9950, 283.2630, 419.2550 701.5130, 788.5410	152.9967, 283.2653, 419.2616, 701.5201, 788.5463	MSBNK-RIKEN_IMS-LQB00333
**834.5**	PS 40:6	152.9950, 283.2643, 419.2550, 747.4940, 834.5290	152.9946, 283.2636, 419.2562, 747.4965, 834.5291	MSBNK-RIKEN_IMS-LQB00340
**885.5**	PI 38:4	241.0120, 283.2630, 303.2320, 419.2550, 581.3090, 885.5470	241.0120, 283.2658, 303.2348, 419.2582, 581.3079, 885.5515	MSBNK-RIKEN_IMS-LQB00308
**888.6**	ST 24:1 (Sulfatide)	NA	96.9605, 241.0038, 390.3767, 888.6271	NA

Table S2 | Table of MS/MS fragments of peaks present in mouse brain experiments. Precursor ion represents the selected mass for MS/MS. Assignment represents the assigned species to the peak. Also shown are the previously reported fragments of the assigned species as well as the observed fragments observed in the measured MS/MS spectra. Lastly are the MassBank Identifier for the reference MS/MS spectra.
